# First-Principles Modelling of Ion-Substituted Hydroxyapatite: Defect Chemistry, Site Preferences, and Structure–Property Relationships—A Critical Review

**DOI:** 10.3390/biomimetics11100686

**Published:** 2026-09-22

**Authors:** Nisa Naseem, Łukasz Szeleszczuk

**Affiliations:** Department of Organic and Physical Chemistry, Medical University of Warsaw, 1 Banacha Str., 02-097 Warsaw, Poland; nisanaseem733@gmail.com

**Keywords:** hydroxyapatite, density functional theory, ion substitution, defect chemistry, charge compensation, site preference, co-doping, surface modelling, biomaterials, machine learning

## Abstract

Hydroxyapatite (HAp) possesses exceptional compositional flexibility, allowing for extensive cationic, anionic, and coupled substitutions that can modify its structural, mechanical, electronic, magnetic, catalytic, and biological properties. However, experimental identification of dopant locations and charge-compensation mechanisms remains challenging, particularly in nanocrystalline, calcium-deficient, and multiphase materials. This review evaluates first-principles studies of ion-substituted HAp, with particular emphasis on substitution-site preferences, defect association, charge compensation, thermodynamic stability, configurational effects, surfaces, hydration, and comparison with experimental observables. Unlike earlier reviews that primarily catalogued individual dopants and their calculated effects, the present work critically examines the methodological and defect-chemical assumptions underlying reported substitution mechanisms, including charge compensation, configurational sampling, chemical-potential definitions, hydration, finite-temperature effects, and experimental validation. The literature demonstrates that dopant site preference is not an intrinsic and concentration-independent property of an element. Instead, it depends on supercell size, dopant concentration, hydroxyl ordering, oxidation and spin states, compensating defects, chemical potentials, hydration, temperature, and the presence of other impurities. Many apparent discrepancies between studies arise from small simulation cells, incomplete configurational sampling, inconsistent formation-energy definitions, or calculations performed for assumed compositions without explicit charge compensation. The strongest structural assignments combine defect energetics with directly calculated spectroscopic parameters. Future predictive models should treat substituted HAp as a condition-dependent ensemble of interacting defects and integrate large-scale configurational sampling, finite-temperature methods, hydrated surfaces, machine learning, and advanced spectroscopic validation.

## 1. Introduction

Hydroxyapatite (HAp, Figure 1), commonly represented by the formula Ca_10_(PO_4_)_6_(OH)_2_, is the principal crystalline model used to describe the inorganic component of mammalian bones and teeth [1,2]. Its chemical similarity to biological apatite, together with its biocompatibility, osteoconductivity, and ability to form direct bonds with mineralized tissues, has led to its widespread use in bone fillers, implant coatings, dental materials, drug-delivery systems, and tissue-engineering scaffolds [2,3]. HAp has also attracted increasing attention in catalysis, environmental remediation, sensing, and bioimaging, where its chemical stability and ion-exchange capacity can be combined with deliberately introduced functional species [4].

A defining feature of the apatite lattice is its exceptional compositional flexibility [1,3]. Calcium ions can be replaced by mono-, di-, tri-, or higher-valent cations, phosphate groups can be substituted by other tetrahedral or planar oxyanions, and the hydroxyl channels can accommodate fluoride, chloride, carbonate, vacancies, or additional protonic defects [1]. These substitutions can modify lattice dimensions, solubility, mechanical response, electronic structure, magnetic properties, luminescence, catalytic activity, and biological performance [1,4]. Natural bone mineral is itself a non-stoichiometric, carbonated and calcium-deficient apatite containing numerous vacancies, protonated phosphate groups, and trace-element substitutions rather than perfectly stoichiometric HAp [1,2].

From a biomimetic perspective, the compositional flexibility of hydroxyapatite is particularly important because biological apatite is not a chemically ideal Ca_10_(PO_4_)_6_(OH)_2_ phase, but a structurally disordered and ion-substituted mineral adapted to the physiological environment [1,2]. Incorporation of ions such as Mg^2+^, Sr^2+^, Zn^2+^, carbonate, or fluoride can therefore be regarded not only as a strategy for engineering synthetic HAp, but also as an attempt to reproduce selected structural and functional features of natural mineralized tissues [1,3]. In this context, first-principles modelling provides an atomistic framework for understanding how biomimetic substitutions, vacancies, and charge-compensation mechanisms modify the local structure and properties of HAp. Such knowledge is essential for the rational design of bone- and tooth-inspired materials whose composition and functionality more closely resemble those of biological apatites.

Experimental methods such as X-ray and neutron diffraction, vibrational spectroscopy, solid-state NMR, EPR, XPS, X-ray absorption spectroscopy, and elemental analysis provide essential information about substituted apatites. Nevertheless, identification of the exact dopant site is often difficult, particularly at low concentrations or in nanocrystalline samples [5,6]. Diffraction-derived site occupancies may be affected by correlated thermal parameters, structural disorder, secondary phases, and limited scattering contrast. Spectroscopic signals may identify the local oxidation state or coordination environment but may not uniquely distinguish between several structurally similar defect models [6]. Furthermore, nominal precursor composition does not necessarily correspond to the amount incorporated into the HAp lattice, as dopants may segregate to surfaces, grain boundaries, amorphous regions, or secondary calcium-phosphate phases.

The problem is especially challenging for aliovalent substitutions. Replacement of Ca^2+^ by Li^+^, Eu^3+^, Ti^4+^, or other heterovalent ions requires charge compensation through calcium or hydroxyl vacancies, protonation or deprotonation, anionic co-substitution, changes in oxidation state, or combinations of these mechanisms [7,8,9]. Different charge-compensation schemes may produce similar average compositions but substantially different local structures, energetics, and properties. For example, calculations for Eu^3+^-HAp have shown that hydroxyl deprotonation and calcium-vacancy compensation lead to different dopant distributions and lattice responses [7].

First-principles calculations provide direct access to the atomistic structures that are difficult to resolve experimentally. Density functional theory can be used to compare substitution sites, charge-compensation mechanisms, defect association, oxidation and spin states, local lattice distortions, electronic structures, elastic properties, surface stability, ion migration, and spectroscopic observables. When combined with experimentally measured lattice parameters, EPR tensors, XPS shifts, vibrational frequencies, or X-ray absorption data, such calculations can discriminate between competing structural models rather than merely rationalize an assumed composition.

Earlier reviews provided useful surveys of computational work on ion-doped HAp [10], but the field has subsequently expanded considerably. Larger supercells, hybrid functionals, DFT+U, chemical-potential-dependent defect thermodynamics, explicit charge-compensation models, surface calculations, machine-learning-assisted configurational searches, and finite-temperature treatments are now increasingly available.

The present review therefore does not aim merely to catalogue individual dopants. Instead, it critically examines the complete sequence from dopant, through substitution site, charge compensation, local geometry, stability, thermodynamic stability, functional properties, and potential applications.

Cationic and anionic substitutions, coupled defects, co-doping, intrinsic vacancies, surfaces, and interfaces are discussed within a unified defect-chemical framework. Particular attention is paid to methodological differences that can explain apparently contradictory site preferences, including supercell size, dopant concentration, functional choice, configurational sampling, hydration, and chemical potentials. This approach distinguishes firmly established substitution mechanisms from electronic-structure calculations performed for assumed compositions and identifies the requirements for genuinely predictive modelling of modified hydroxyapatites.

## 2. Literature Search and Study Selection

Relevant peer-reviewed studies were identified through a structured literature search in Scopus, supplemented by Google Scholar, backward reference screening, and forward citation tracking of key publications. Studies were considered when first-principles calculations constituted a substantive component and addressed ionic substitution, intrinsic defects, charge compensation, co-doping, surfaces, or related structure–property relationships in hydroxyapatite. Purely experimental studies and reports based exclusively on classical simulations were excluded unless they provided essential mechanistic context for a DFT-validated model. Two reviewers (N.N. and Ł.S.) independently evaluated the relevance of the identified studies, and disagreements were resolved by consensus.

## 3. Crystal Structure and Defect Chemistry of Hydroxyapatite

### 3.1. Hexagonal and Monoclinic Structures

Stoichiometric hydroxyapatite is most commonly described by the hexagonal space group $P6_{3}/m$, with lattice parameters of approximately a = b = 9.4 Å and c = 6.9 Å. The conventional hexagonal unit cell contains two Ca_5_(PO_4_)_3_OH formula units, corresponding to 44 atoms: ten Ca atoms, six PO_4_ tetrahedra, and two hydroxyl groups. The phosphate tetrahedra form the principal structural framework, while calcium ions connect adjacent phosphate groups and surround channels extending parallel to the crystallographic c-axis.

The centrosymmetric *P*6_3_/m structure represents an average arrangement in which the orientations of OH groups are disordered between symmetry-related positions. When the hydroxyl dipoles become ordered, HAp can adopt a monoclinic structure, most commonly described using the *P*2_1_/b space group. The monoclinic cell contains 88 atoms and permits an antiparallel arrangement of the hydroxyl groups. A polar *P*2_1_ arrangement, in which the hydroxyl dipoles are aligned in one direction, has also been considered computationally in connection with possible piezoelectric and ferroelectric-like properties. The relative occurrence of these structures depends on stoichiometry, impurity content, temperature, and hydroxyl ordering.

This structural distinction is important in computational studies. A hexagonal model may impose artificial symmetry between apparently equivalent substitution sites, whereas hydroxyl ordering in a monoclinic model can split these sites into locally nonequivalent environments. Consequently, a dopant found to have no site preference in a high-symmetry unit cell may display a measurable preference when the hydroxyl orientation and local defect configuration are treated explicitly.

### 3.2. Calcium Sites and the Hydroxyl Channel

The HAp lattice contains two crystallographically distinct calcium sublattices, conventionally designated Ca(I) and Ca(II). Ca(I) ions form columns parallel to the c-axis and are coordinated exclusively by oxygen atoms belonging to phosphate groups. Depending on the distance criterion, their environment may be described as six strongly bound oxygen atoms with three more distant contacts or as an irregular nine-coordinate polyhedron.

Ca(II) ions form triangular arrangements around the hydroxyl channel. Their first coordination shell is usually described as containing seven oxygen atoms, six derived from phosphate groups and one from a hydroxyl group. The resulting geometry resembles a distorted pentagonal bipyramid.

The distinction between Ca(I) and Ca(II) is therefore chemical rather than merely crystallographic. Substitution at Ca(I) primarily perturbs phosphate tetrahedra and calcium columns. Substitution at Ca(II) can additionally interact directly with the hydroxyl channel and may induce hydroxyl displacement, deprotonation, channel contraction, or local reorientation. This explains why dopants with comparable ionic radii may nevertheless exhibit different site preferences and why the preferred site can change when the channel composition is modified.

The hydroxyl groups form one-dimensional chains along the c-axis. These channels can accommodate F^−^, Cl^−^, carbonate, oxygen species, vacancies, and protonic defects. Replacement of OH^−^ by F^−^ is formally isovalent, whereas hydroxyl removal may compensate a heterovalent substitution elsewhere in the lattice. The channel also provides possible pathways for proton or hydroxyl migration under thermal, electrical, or non-equilibrium conditions.

### 3.3. Substitutional and Intrinsic Defects

Cationic substitution most frequently occurs at Ca(I) or Ca(II). Isovalent ions such as Mg^2+^, Zn^2+^, Sr^2+^, Cd^2+^, and Pb^2+^ can replace Ca^2+^ without formal charge compensation, although they generate elastic and coordination strain. Aliovalent cations require additional defects. Substitution of Ca^2+^ by a monovalent ion introduces a negative effective charge, whereas trivalent and tetravalent cations introduce one or two positive effective charges, respectively.

The phosphate site can accommodate oxyanions such as SiO_4_^4−^, CO_3_^2−^, NO_3_^−^, AsO_4_^3−^, and VO_4_^3−^. Replacement of PO_4_^3−^ by an anion of different charge must be coupled to protonation, cation vacancies, hydroxyl vacancies, or co-substitution. Carbonate can occur as B-type carbonate replacing PO_4_^3−^ or as A-type carbonate occupying the hydroxyl channel. The two configurations have different charge-compensation requirements and different structural consequences.

Intrinsic defects include calcium vacancies, hydroxyl vacancies, hydrogen vacancies or deprotonated hydroxyl groups; oxygen vacancies within phosphate groups or channel sites; H, OH, and selected cation interstitials.

Such defects can introduce localized electronic states, modify proton transport, change surface charge, and influence dopant incorporation. First-principles studies have demonstrated that O, H, and OH vacancies produce distinct electronic states and structural responses rather than representing interchangeable descriptions of oxygen deficiency [5].

Protonation of PO_4_^3−^ to HPO_4_^2−^ is another important compensation mechanism. It is particularly relevant to calcium-deficient and biologically derived apatites. For example, replacement of Ca^2+^ by Li^+^ can be compensated either by eliminating an OH^−^ group or by protonating a phosphate group, and the two models lead to different local distortions and energetics [8].

### 3.4. Stoichiometric and Calcium-Deficient Hydroxyapatite

Ideal stoichiometric HAp has a Ca/P ratio of 1.67. Synthetic precipitates and biological apatites commonly possess lower Ca/P ratios and are better represented as calcium-deficient hydroxyapatite. A frequently used general formula is

$$Ca_{10-x}{\left(HPO_{4}\right)}_{x}{\left(PO_{4}\right)}_{6-x}{\left(OH\right)}_{2-x},$$

where 0 < x ≤ 1. Calcium deficiency is compensated through a coupled increase in HPO_4_^2−^ content and a decrease in hydroxyl occupancy. The limiting value x=1 corresponds to a Ca/P ratio of 1.50.

This expression represents an idealized defect scheme rather than a unique structure. Real calcium-deficient HAp may additionally contain carbonate, structural water, sodium, magnesium, adsorbed ions, amorphous calcium phosphate, and surfaces enriched in protonated phosphate groups. Calcium vacancies and protonated phosphate units may be associated locally or distributed across the crystal.

The distinction between stoichiometric and calcium-deficient HAp is crucial when interpreting dopant calculations. A dopant that is energetically unfavorable in perfect Ca_10_(PO_4_)_6_(OH)_2_ may be stabilized by pre-existing calcium deficiency. Conversely, a nominally “doped HAp” sample may actually consist of a calcium-deficient apatite, a secondary calcium-phosphate phase, and surface-associated dopant species. The stoichiometric crystal should therefore be regarded as a well-defined reference state, not as a complete atomistic representation of biological apatite.

## 4. Computational Strategies for Modelling Doped Hydroxyapatites

### 4.1. Unit Cells, Supercells, and Periodic Models

Periodic density functional theory is the most widely used method for modelling crystalline HAp because it describes the extended ionic lattice without artificial molecular boundaries. However, the selected cell size determines both the nominal dopant concentration and the interaction between periodic defect images.

The 44-atom hexagonal unit cell contains ten calcium, six phosphorus, and two hydroxyl sites. Consequently, replacement of one Ca atom corresponds to 10% substitution of the calcium sublattice, replacement of one phosphate group corresponds to 16.7% of the phosphorus sites, and substitution of one hydroxyl group represents 50% of the channel sites. Such concentrations are considerably higher than those of many experimental materials.

The 88-atom monoclinic cell reduces a single calcium substitution to 5%, while a 2 × 2 × 2 replication of the hexagonal cell contains 352 atoms and 80 Ca sites, allowing concentrations as low as 1.25% to be modelled explicitly. The latter approach has been used successfully for low-concentration Zn and Eu substitutions [7,11].

Small cells may exaggerate dopant–dopant and defect–defect interactions, enforce artificial ordering, suppress local symmetry breaking, and prevent formation of realistic dilute configurations. They can nevertheless remain useful for screening alternative oxidation states or compensation mechanisms, provided that the resulting concentration is reported explicitly.

### 4.2. Periodic DFT and Cluster Approaches

Periodic plane-wave calculations are well suited to lattice optimization, defect energetics, band structures, elastic properties, phonons, surfaces, and migration pathways. They also permit chemically meaningful comparison of structures containing extended strain fields.

Cluster models describe a finite fragment of the HAp lattice using localized atomic orbitals. They can be valuable for detailed bonding analysis, high-level electronic-structure calculations, core-level spectroscopy, and local magnetic parameters. However, the fragment boundaries must be terminated or embedded carefully, and the selected charge and multiplicity may dominate the result. An isolated gas-phase cluster cannot automatically reproduce long-range electrostatics, lattice strain, or the relative stability of Ca(I) and Ca(II) sites in the crystal.

Embedded clusters, quantum-mechanical/molecular-mechanical models, and periodic calculations should therefore be regarded as complementary. Cluster calculations become particularly convincing when the local geometry is extracted from a periodic model and the surrounding lattice is represented by point charges or an embedding potential.

### 4.3. Exchange–Correlation Functionals and Electronic Structure

LDA, PW91, PBE, and PBEsol have all been used for modified HAp. LDA commonly produces shorter bonds and smaller cell volumes, whereas PBE and PW91 tend to overestimate lattice parameters slightly. PBEsol often improves structural properties of densely packed solids but may not necessarily provide better defect energies.

Hybrid functionals such as HSE06 and B3LYP include a fraction of exact exchange and generally provide more realistic band gaps and localization of defect states. Their computational cost restricts their use in large supercells, and a common strategy is to optimize structures with a semilocal functional and perform hybrid-functional single-point calculations for selected configurations.

The selected functional is particularly important when a paper interprets changes in conductivity, optical absorption, or photocatalysis from the calculated band gap. A reduction in the Kohn–Sham gap does not by itself imply the presence of mobile charge carriers. The location, occupancy, and localization of dopant-derived states must also be examined.

For transition-metal and rare-earth dopants, spin polarization is essential. Calculations performed in a non-spin-polarized state may produce qualitatively incorrect structures and electronic levels. Different oxidation and spin states should be tested rather than assumed from the precursor compound.

DFT+U can improve localization of partially occupied d or f states, but the result depends on the selected U parameter. Whenever possible, U should be derived from linear response or validated against spectroscopic data. Hybrid functionals provide an alternative but do not eliminate the need to test alternative spin states.

### 4.4. Pseudopotentials, Basis Sets, and Dispersion

Plane-wave calculations generally employ norm-conserving, ultrasoft, or PAW pseudopotentials. Cutoff energies and k-point meshes must be converged for the specific pseudopotentials rather than adopted directly from earlier work. Heavy dopants may require scalar-relativistic treatments and inclusion of semicore states. For rare-earth elements, authors should state explicitly whether the 4f electrons are treated as valence states or frozen into the core.

Particular caution is required when site preferences are inferred from very small total-energy differences. Numerical convergence of each individual calculation does not necessarily imply convergence of the energy difference between two competing defect configurations. Several studies reviewed here report plane-wave cutoffs, k-point meshes, SCF thresholds, and geometry-optimization criteria, but systematic convergence tests of the site-preference energy itself are rarely presented. For example, the Mg-HAp study reporting Ca(I)–Ca(II) differences of only 0.012 and 0.077 eV employed a 340 eV plane-wave cutoff, a 3 × 3 × 4 k-point mesh, and tight electronic and structural convergence criteria, but did not report how these small energy differences changed upon further increase in the cutoff or k-point density or upon changing the exchange–correlation functional [12].

More generally, energy differences on the order of approximately 0.01–0.1 eV should not automatically be interpreted as robust evidence of exclusive site preference. At this scale, the predicted ordering may be comparable to residual numerical uncertainty or to changes introduced by the functional, pseudopotential, supercell size, hydroxyl ordering, and structural relaxation protocol. A convincing assignment therefore requires convergence of the energy difference itself, rather than only of the absolute total energies. When such tests are unavailable, terms such as “weak preference”, “near-degenerate configurations”, or “possible mixed occupancy” are more appropriate than an unequivocal assignment to a single lattice site.

Localized-orbital calculations require comparable attention to basis-set completeness, basis-set superposition error, and the quality of diffuse or polarization functions. A large nominal basis does not compensate for an unrealistic cluster boundary or charge state.

Dispersion corrections generally exert a limited effect on the ionic bulk structure of stoichiometric HAp. They become more relevant for organic molecules, polymers, proteins, graphene derivatives, molecular adsorbates, and interfaces. Comparisons of adsorption or binding energies should therefore use the same dispersion treatment for the clean surface, adsorbate, and combined system.

### 4.5. Charge Compensation and Charged Defects

The most reliable models preserve charge neutrality by introducing explicit chemical defects. Thus, Eu^3+^ substitution should be accompanied by hydroxyl deprotonation or calcium vacancies, Li^+^ by phosphate protonation or hydroxyl loss, and SiO_4_^4−^ by a hydroxyl vacancy or protonated silicate. The location of the compensating defect must also be sampled because adjacent and separated configurations may differ substantially in energy.

When an isolated charged defect is modelled, its formation energy can be expressed as

$$E_{f}\left(D^{q}\right)=E_{tot}\left(D^{q}\right)-E_{tot}\left(HAp\right)-\sum_{i}n_{i}\mu_{i}+q\left(E_{F}+E_{VBM}+\Delta V\right)+E_{corr},$$

where $n_{i}$ and $\mu_{i}$ describe the numbers and chemical potentials of exchanged species, q is the defect charge, $E_{F}$ is the Fermi level, ΔV aligns the electrostatic potentials, and $E_{corr}$ corrects finite-size interactions.

Periodic charged cells require a compensating background and finite-size corrections because the defect interacts artificially with its periodic replicas. Makov–Payne-type and more advanced correction schemes should be considered, especially in small or anisotropic cells [6].

In many HAp studies, explicit neutral defect complexes are preferable because they avoid some of these complications and often correspond more closely to the actual defect chemistry. The reviewed models are classified as neutral isovalent substitutions, neutral compensated defect complexes, explicitly charged defects treated with finite-size corrections, or chemically uncompensated/assumed compositions for which no explicit charged-defect correction was reported. This classification refers to the actual computational treatment of the periodic model rather than solely to the formal oxidation state assigned to the dopant. A representative example of an explicitly charged-defect formalism is the Fe-HAp study of Avakyan et al. [6], in which charged supercells were treated using a compensating background and a Lany–Zunger-type finite-size correction.

### 4.6. Substitution, Incorporation, and Formation Energies

The terms substitution energy, incorporation energy, and formation energy are not used consistently in the literature. A simple cation-substitution reaction may be represented by

$$E_{sub}=E\left(HAp:M\right)+\mu_{Ca}-E\left(HAp\right)-\mu_{M}.$$

Its value depends entirely on the definitions of $\mu_{Ca}$ and $\mu_{M}$. Metallic elements, isolated atoms, oxides, hydroxides, aqueous ions, and phosphate salts constitute different reservoirs and can change the numerical result by several electron volts.

Chemical potentials should be constrained by the stability of HAp and competing phases, including CaO, Ca(OH)_2_, calcium phosphates, and relevant dopant oxides or phosphates. A sophisticated example is provided by Fe-HAp, for which Ca-rich, Ca-poor, P-rich, and P-poor conditions were examined explicitly. Calcium substitutions were favored under Ca-poor conditions, whereas interstitial Fe near the hydroxyl channel became more favorable under Ca-rich conditions [6].

Therefore, formation energies reported in different publications should not be compared quantitatively unless the reactions, compositions, reservoirs, and sign conventions are equivalent. Relative energies between configurations of identical composition are considerably more robust.

For co-doping, a pair-interaction energy can be defined as

$$E_{int}=E\left(HAp:A+B\right)+E\left(HAp\right)-E\left(HAp:A\right)-E\left(HAp:B\right).$$

A negative value demonstrates energetic association only when all four calculations use the same supercell, functional, basis, and defect stoichiometry.

### 4.7. Dopant Concentration and Configurational Sampling

At a fixed composition, numerous configurations may differ in the occupied Ca sublattice, dopant separation, hydroxyl orientation, and location of compensating vacancies. Optimizing one arbitrarily selected structure does not establish the global minimum.

This problem becomes severe for multiple dopants. A machine-learning-assisted study of Zn-HAp generated approximately 4000 DFT-optimized structures and showed that configurations with identical Zn concentration could differ by tens of kilocalories per mole. Even among models in which Zn occupied only Ca(II), relative placement of several Zn ions produced differences of up to 24.4 kcal mol^−1^ [11].

Configurational enumeration, symmetry reduction, stochastic sampling, cluster expansion, and machine-learning potentials offer more systematic alternatives to manual placement. Machine learning should primarily act as a search accelerator; candidate low-energy configurations should be re-evaluated using the underlying first-principles method.

At finite temperature, the relevant structure may be an ensemble rather than a unique ground state. For Sr-HAp, inclusion of configurational entropy changed the predicted distribution from a slight Ca(I) preference at 0 K to mixed Ca(I)/Ca(II) occupancy under experimentally relevant conditions [13].

### 4.8. Surfaces and Aqueous Environments

Bulk substitution energies cannot be transferred directly to surfaces. Surface models require selection of crystallographic orientation, termination, slab thickness, vacuum spacing, stoichiometry, and compensation of dipoles. The two sides of a slab should be equivalent or corrected appropriately, and the relaxed structure should be checked for interactions between periodic images.

Vacuum calculations may produce substantial reconstruction of exposed calcium, phosphate, and hydroxyl groups. Implicit-solvent approaches account for dielectric screening but cannot describe discrete hydrogen bonding, proton transfer, or ion hydration. Explicit water captures local solvation but requires larger cells and configurational or molecular-dynamics sampling.

Hydration can change not only adsorption energies but also bulk defect stability. For Si-HAp, the preferred mechanism shifted from hydroxyl-vacancy compensation under dehydrating conditions to protonated HSiO_4_ under water-rich conditions [14].

Similarly, ideal surface calculations found that Si substitution altered surface electrostatics but did not substantially reduce the barrier for Ca removal, suggesting that experimentally increased dissolution may arise from grain size, disorder, and surface area rather than an intrinsic weakening of one ideal surface site (Figure 2) [15].

### 4.9. Vibrational and Finite-Temperature Stability

Geometry optimization establishes only a stationary point on the potential-energy surface. Phonon calculations are required to verify dynamical stability and to identify unstable distortions, hydroxyl-ordering modes, or defect-induced soft modes. Vibrational free energies may also modify small differences between competing configurations.

Ab initio molecular dynamics can examine hydroxyl reorientation, proton transfer, local hydration, dopant coordination, and structural stability at finite temperature. It is particularly valuable for surfaces and highly defective structures, although accessible simulation times remain short relative to crystallization and dissolution processes.

Configurational entropy, vibrational contributions, hydration, and thermal expansion are rarely treated simultaneously in current studies of doped HAp. As a result, most reported site preferences should be interpreted as 0 K predictions for a particular ordered configuration rather than direct predictions of experimental occupancies.

Overall, a conclusion that a dopant “prefers” Ca(I), Ca(II), a phosphate site, or the hydroxyl channel is conditional on the adopted model. It may change with supercell size, dopant concentration, hydroxyl ordering, charge-compensation mechanism, oxidation and spin state, hydration, chemical potentials, configurational entropy, and the presence of competing defects or phases. Predictive modelling therefore requires not only an accurate electronic-structure method but also a chemically realistic definition of the defect being calculated.

## 5. Cationic Substitution in Hydroxyapatite

Cationic substitution is one of the most extensively investigated routes for modifying hydroxyapatite (HAp), owing to the ability of its calcium sublattice to accommodate ions that differ substantially in size, electronegativity, oxidation state, and electronic configuration. The two crystallographically nonequivalent calcium sites, conventionally denoted Ca(I) and Ca(II), provide chemically distinct coordination environments. Ca(I) sites form columns parallel to the crystallographic c-axis and are coordinated exclusively by phosphate oxygen atoms, whereas Ca(II) sites surround the hydroxyl channel and include an oxygen atom originating from an OH group in their first coordination sphere. Consequently, incorporation into Ca(II) may directly perturb the hydroxyl channel, while substitution at Ca(I) primarily affects the arrangement of phosphate tetrahedra and cationic columns.

The accumulated computational evidence demonstrates that site preference cannot be treated as an intrinsic and concentration-independent property of a dopant. Instead, the preferred arrangement emerges from a combination of ionic size, local coordination, metal–oxygen bonding, hydroxyl ordering, dopant concentration, charge-compensating defects, and configurational entropy. This distinction becomes particularly important when more than one dopant is present. For Zn-HAp, for example, configurations containing identical numbers of Zn ions at nominally equivalent Ca(II) sites can differ by more than 20 kcal mol^−1^ because of the relative positions of the dopants [11]. Similarly, calculations for Sr-HAp show that a weak Ca(I) preference obtained from 0 K energies is replaced by a predominantly mixed Ca(I)/Ca(II) distribution once the configurational entropy characteristic of experimental synthesis temperatures is included [13]. Thus, comparisons based on one arbitrarily selected Ca(I) and one Ca(II) configuration provide, at best, an incomplete description of cation incorporation.

### 5.1. Isovalent Substitution by Divalent Cations

#### 5.1.1. Magnesium

Magnesium is considerably smaller than Ca^2+^ and therefore produces a characteristic contraction of its local oxygen coordination environment. Periodic PBE calculations for 5 and 10% Mg substitution predicted Ca(I) to be slightly more favorable than Ca(II), but the respective energy differences were only 0.012 and 0.077 eV. These values are sufficiently small that they should be regarded as near-degenerate within the methodological resolution of the calculations rather than as evidence for an exclusive Ca(I) preference; both sites may therefore be populated under realistic conditions. The calculated average Mg(I)–O distances were approximately 13.5% shorter than the corresponding Ca(I)–O distances, illustrating that much of the structural response remains localized around the dopant [12].

The experimentally achievable level of Mg incorporation is often substantially lower than the concentrations imposed in unit-cell calculations. In one combined experimental and computational study, only approximately 5–7 mol.% Mg was successfully incorporated into HAp, with increasing Mg content reducing crystallinity, crystallite size, lattice parameters, and thermal stability [12]. Other calculations using larger supercells found no decisive Mg site preference, while hybrid-functional models reported nearly degenerate low-energy arrangements [16,17]. The literature therefore supports a weak tendency toward Ca(I), rather than an exclusive occupation of this site.

The structural consequences of Mg substitution are more consistent than the predicted site occupancies. The smaller Mg^2+^ ion contracts the lattice, reduces the coordination number in some Ca(II)-based models, and generally increases local distortion. These effects have been associated with changes in elastic properties and increased resistance to compression, although the magnitude of the predicted mechanical response depends strongly on dopant concentration and the selected configuration. In comparative calculations, Mg-HAp was predicted to be more resistant to compression than both pristine and Sr-substituted HAp [18].

#### 5.1.2. Zinc

Most periodic calculations indicate a preference of Zn^2+^ for Ca(II), although the magnitude of this preference depends on the structural model. The tendency has been attributed to the smaller size of Zn^2+^, its stronger and partly covalent interactions with oxygen, and the ability of the Ca(II) environment to undergo local relaxation toward shorter Zn–O distances. Importantly, Zn and Cd were predicted to prefer Ca(II) in hydroxyapatite but Ca(I) in fluorapatite, demonstrating that site preference depends not only on the dopant but also on the chemistry and ordering of the anionic channel [19].

A particularly comprehensive treatment combined DFT with a convolutional neural network to explore Zn concentrations from 1.25 to 6.25 mol.% in a 2 × 2 × 2 HAp supercell containing 80 calcium sites. An iterative DFT–machine-learning workflow generated 4000 configurations and used the trained model to identify additional structures near the low-energy region of the potential-energy surface [11]. The first Zn ion preferentially occupied Ca(II), while subsequent Zn ions also entered Ca(II) sites in an approximately symmetric manner. This arrangement reduced both Zn–Zn interactions and global lattice deformation [11].

This work provides an important refinement to the conventional concept of site preference. Once several Zn ions are introduced, the relevant question is no longer simply whether Ca(I) or Ca(II) is favored. The distribution of Zn among different Ca(II) triangles and hydroxyl channels becomes equally important. Limited or random selection of configurations may fail to locate the global low-energy region even at relatively low dopant concentrations. Although the CNN achieved a mean absolute error of approximately 0.14 eV, final low-energy structures were re-evaluated using DFT, appropriately treating machine learning as a configurational-screening tool rather than as a replacement for first-principles calculations [11].

Zn substitution usually contracts the unit cell and alters its mechanical properties. Calculated stress–strain relationships indicate that suitably distributed Zn ions may improve fracture toughness. Nevertheless, experimentally observed improvements in biological or mechanical performance cannot automatically be assigned to bulk substitution alone, as Zn may also influence crystallinity, secondary-phase formation, surfaces, and interactions with organic components.

#### 5.1.3. Strontium

Strontium provides one of the clearest demonstrations that 0 K substitution energies do not necessarily predict experimental site occupancies. Because Sr^2+^ is larger than Ca^2+^, its incorporation produces an approximately linear expansion of both *a* and *c* lattice parameters across a broad compositional range. Experimental Rietveld analyses have nevertheless produced apparently contradictory site preferences. Ca(I) may be favored at very low Sr contents, approximately statistical occupancy is observed at intermediate concentrations, and Ca(II) becomes progressively enriched at higher concentrations. In one study, the Sr(II)/Sr(I) occupancy ratio increased from approximately statistical behavior at 5 at.% Sr to 1.80 and 1.98 at 10 and 15 at.%, respectively [20].

Periodic calculations for ordered structures at 0 K generally predict a small energetic advantage for Ca(I). Accommodation of Sr at Ca(I) can occur through coordinated displacement of the neighboring PO_4_ tetrahedra, whereas Ca(II) occupation may produce stronger distortions in cation–cation distances and metaprismatic twist angles. However, the calculated differences between numerous configurations are often only a few kilojoules per mole [20].

The apparent disagreement with experiment is substantially resolved by including configurational entropy. Free energies of mixing were calculated for preferential Ca(I), preferential Ca(II), and mixed substitution at 90, 700, and 950 °C. Although Ca(I)-first substitution was less energetically demanding at 0 K, the entropy term stabilized mixed occupation at typical calcination and fictive temperatures. Mixed distribution also remained slightly favorable for wet-chemical synthesis, except near 20% Sr, where Ca(II)-rich and mixed arrangements became nearly equivalent [13].

At 50% substitution, the lowest-energy configuration contained two Sr ions at Ca(I) and three at Ca(II), while several other mixed configurations were located within approximately 3 kJ mol^−1^ [13]. Accordingly, experimentally measured site occupancies should be understood as thermally and configurationally averaged distributions rather than as direct manifestations of a single 0 K ground-state structure.

Sr substitution generally decreases elastic moduli moderately while increasing structural and mechanical anisotropy. Its influence on the electronic band gap is comparatively small because Sr^2+^ is isovalent with Ca^2+^ and does not normally create strongly localized states near the band edges. Such relatively subtle modifications are advantageous in dental and bone-filler applications, where Sr can tune mechanical and optical matching without fundamentally changing the insulating character of HAp [21].

#### 5.1.4. Cadmium and Lead

Although Cd^2+^ and Pb^2+^ are biologically undesirable, their incorporation into HAp is relevant to understanding heavy-metal accumulation in mineralized tissues and the use of apatites as environmental sorbents.

For Cd-HAp, synchrotron XRD and Rietveld refinement revealed no resolvable preference at 1.2 at.% Cd but a clear enrichment of Ca(II) at 13 and 25 at.% [22]. Periodic calculations using 88-atom supercells and multiple configurations similarly supported Ca(II) occupation. Embedded-cluster analyses suggested that the preference cannot be explained solely by ionic radius and arises partly from stronger Cd–O and Cd–OH interactions [22]. The progressive shift and broadening of the OH stretching band with increasing Cd content provides experimental evidence that Cd strongly perturbs the hydroxyl channel.

Lead also preferentially occupies Ca(II) at low and intermediate concentrations. However, the predicted distribution changes with composition: Ca(II)-dominated configurations were favored up to approximately 40% Pb, whereas mixed Ca(I)/Ca(II) arrangements became more stable at 50–60% substitution [23]. The Pb-HAp system therefore reinforces the conclusion that a site preference determined in the dilute limit should not be extrapolated over the entire solid-solution range.

### 5.2. Transition-Metal Substitution and Oxidation-State Effects

Transition-metal dopants introduce additional complexity because their partially occupied *d* shells may generate localized defect states, magnetic moments, multiple oxidation states, and spin-dependent properties.

For manganese, theoretical and experimental results vary with concentration and the observable used to identify the preferred site. In the concentrated periodic models of Bystrov et al. [24], structural optimization was first performed using GGA-PBE and the electronic structure was subsequently refined using the HSE/HSE06 hybrid functional; no Hubbard U correction was employed. These spin-polarized calculations predicted Ca(II) occupation and the formation of Mn-derived states within the HAp band gap. Increasing Mn content reduced the lattice volume and increased the calculated magnetization [24]. In contrast, the highly dilute Mn^2+^ study of Shurtakova et al. [25] used conventional GGA-PBE without DFT+U or a hybrid functional and combined the calculated electron density with EPR analysis.

The latter study calculated crystal-field parameters from the full DFT electron density rather than from a point-charge approximation. The strongly nonspherical electron density of the neighboring PO_4_ groups made the point-charge model inadequate, particularly for higher-order crystal-field terms [25]. The apparent difference between energetic and spectroscopic studies is therefore not necessarily contradictory: it may reflect differences in concentration, synthesis history, configurational averaging, and the distinction between a lowest-energy ordered model and the distribution of impurity centers present in an experimental powder.

Iron illustrates the importance of explicitly distinguishing both oxidation states and the electronic-structure treatment. The Fe^2+^/Fe^3+^ site-preference study of Makshakova et al. [26] employed periodic GGA-PBE calculations without either a Hubbard U correction or a hybrid functional. Fe^2+^ is an isovalent substitute for Ca^2+^, whereas Fe^3+^ requires charge compensation. In that model, Fe^2+^ could occupy both Ca sites, with only an approximately 0.2 eV preference for Ca(II), whereas Fe^3+^ compensated by hydroxyl deprotonation exhibited a stronger Ca(II) preference of approximately 1.03 eV and produced greater lattice contraction and shorter Fe–O bonds [26]. By contrast, the more extensive charged-defect study of Avakyan et al. [6] employed spin-polarized HSE hybrid DFT rather than DFT+U to describe substitutional and interstitial Fe defects and their charge states. Consequently, quantitative energetic and electronic comparisons between these Fe studies should take the different exchange-correlation treatments into account.

For Co-, Ni-, and Cu-substituted HAp, Sadetskaya et al. [27] compared conventional LDA and GGA calculations using ABINIT; neither DFT+U nor a hybrid functional was employed. The authors reported that LDA gave band-gap values closer to experiment than GGA within their modelling framework. Because localized 3d states were therefore treated without an explicit Hubbard correction or exact exchange, the predicted positions of dopant-derived states and the resulting band gaps should be interpreted cautiously. Moreover, the study used predefined substitutional models and did not explore the complete configurational or spin-state landscape. Its results are consequently more informative regarding electronic-structure trends than regarding the thermodynamic mechanism of incorporation [27]. At higher Co contents, experimental systems may depart from substitutional solid solutions, with loss of HAp ordering and formation of cobalt-containing surface nanoparticles [28]. The accompanying DFT calculations in that study employed the semilocal PBE functional without a reported Hubbard U correction or hybrid-functional treatment.

### 5.3. Aliovalent Cations and Charge-Compensating Defects

#### 5.3.1. Lithium

Substitution of Ca^2+^ by Li^+^ introduces a negative effective charge and therefore requires a positively charged compensating defect. Two mechanisms have been compared directly: removal of an OH^−^ group from the hydroxyl channel and protonation of PO_4_^3−^ to form HPO_4_^2−^. Periodic PBE calculations found that Li^+^ preferred Ca(I) by approximately 0.2 eV for both compensation routes [8].

Li occupation of Ca(I) produced relatively limited structural distortion, whereas insertion into Ca(II), adjacent to the hydroxyl channel, caused substantial channel deformation. EPR detection of isolated hydrogen-related centers was interpreted as support for compensation involving hydroxyl loss [8]. The Li-HAp system is therefore a particularly clear example of how substitution-site and compensation-mechanism preferences should be evaluated simultaneously.

#### 5.3.2. Aluminum

Al^3+^ substitution was modeled using the calcium-deficient composition Ca_10−1_._5x_Al_x_(PO_4_)_6_(OH)_2_, in which two Al^3+^ ions replacing Ca^2+^ are compensated by a calcium vacancy. Eight combinations of Al and vacancy positions were compared. The most stable model contained Al at Ca(II) and the calcium vacancy at a neighboring Ca(II) site [29].

Relaxation caused displacement of Al from the original Ca position, rotation and deformation of phosphate tetrahedra, and disruption of the OH arrangement [29]. This result demonstrates that the relevant defect is not an isolated Al substitution but an Al–vacancy complex with its own preferred geometry.

The proposed connection between the increased total energy of Al-HAp and enhanced osteoclastic dissolution should nevertheless be treated cautiously. Bulk static calculations do not directly model hydrated surfaces, acidic cellular environments, dissolution kinetics, or biological resorption [29]. They establish relative structural destabilization of a selected defect model but not a complete mechanism of Al-related bone disease.

#### 5.3.3. Trivalent Rare-Earth Ions

Trivalent rare-earth dopants formally introduce one positive effective charge for each Ca^2+^ ion replaced. Accordingly, meaningful atomistic models require calcium vacancies, hydroxyl deprotonation, anionic substitutions, or other coupled defects.

A comprehensive Eu^3+^ study compared compensation by calcium vacancies and by deprotonation of OH groups in a 352-atom model. Deprotonation was energetically more favorable and promoted a more uniform distribution of Eu, whereas calcium-vacancy compensation encouraged locally clustered defect complexes. Eu showed a moderate preference for Ca(II), but the calculated differences were sufficiently small that both sites could be occupied at experimental synthesis temperatures [7].

By contrast, many studies of La-, Sm-, Dy-, and Pr-containing HAp assume a selected Ca site and proceed directly to band-structure and density-of-states calculations. In some cases, occupation of Ca(II) is described as “preferred” even though Ca(I) was not explicitly evaluated. Calculations performed without spin polarization are additionally problematic for open-shell rare-earth ions, while semilocal functionals without an appropriate treatment of localized *f* electrons may place dopant states incorrectly [30].

Reported reductions in band gap for Dy- or Pr-containing structures should therefore be interpreted as properties of the assumed periodic models, rather than as definitive predictions for experimentally synthesized defect ensembles. For highly substituted Pr-HAp, experimental calcium deficiency suggests vacancy-based compensation, but equivalent vacancies were not always included in the electronic-structure models [31].

#### 5.3.4. Titanium and Other High-Valence Cations

Ti incorporation demonstrates that the chemical form and charge-compensation scheme of the substituting species can be more important than its nominal elemental identity. Yin and Ellis [9] considered three distinct Ti-containing models. In the first, direct replacement of Ca^2+^ by bare Ti^4+^ corresponds formally to an effective defect charge of +2 relative to the host Ca site. This chemically uncompensated model produced nearly equivalent Ca(I) and Ca(II) total energies and was used primarily to examine the structural response to direct Ti incorporation. In the second model, Ti^4+^ substitution was compensated by removal of two protons from hydroxyl groups, yielding an overall charge-neutral defect complex. This compensation changed the energetic ordering substantially, making Ca(II) more favorable by approximately 2.0 eV because channel oxygen atoms could move toward Ti and form short Ti–O bonds. In the third model, Ti was incorporated as the formally divalent Ti(OH)_2_^2+^ species, which is isovalent with Ca^2+^ and therefore also forms an overall charge-neutral substitutional model. Ti(OH)_2_^2+^ showed a strong preference for Ca(II), with several Ca(II) configurations being thermodynamically favorable relative to the selected reservoirs [9].

The principal calculations for all three models employed one Ti-containing species in a 1 × 1 × 1 HAp unit cell, corresponding to approximately 10 at.% Ti. However, their thermodynamic reference states were not fully equivalent. For the charge-compensated Ti^4+^ model, the substitution reaction was defined using Ti(OH)_4_ and Ca(OH)_2_ together with two H_2_O molecules, whereas the Ti(OH)_2_^2+^ substitution used Ti(OH)_4_ and Ca(OH)_2_ without the H_2_O term. The uncompensated bare-Ti model was not assigned a directly comparable substitution energy. Consequently, absolute substitution energies for the three models should not be compared as though they were defined on an identical chemical-potential scale. As an additional concentration check, Ti(OH)_2_^2+^ was also examined at 5 at.% in a 1 × 1 × 2 supercell, where the preference for Ca(II) remained strong, although the site-energy difference decreased to approximately 1.5 eV [9].

A more recent defect study similarly found that calcium vacancies facilitate Ti^4+^ incorporation and lower the energetic cost of forming Ti-associated defect structures. Zn^2+^, by comparison, remained an isovalent dopant with a modest Ca(II) preference. The absolute formation energies in that work depend on the selected elemental reservoirs, and conclusions regarding spontaneous vacancy formation should therefore be linked explicitly to the assumed chemical potentials.

The Ti models commonly contain one substitution in a unit cell, corresponding to approximately 10 at.% Ti. Such concentrations amplify interactions between periodic defect images and may not represent dilute experimental samples; the original authors themselves noted that larger supercells are required to study lower concentrations [9].

Nb-containing studies require still greater caution. Some calculations were conducted for 44-atom molecular fragments in the gas phase using Gaussian and mixed molecular basis sets rather than a periodic crystalline model. Experimental evidence also indicated β-TCP and niobium-oxide-related phases, while alternative replacement of phosphate groups by niobate species was considered [32]. Such calculations cannot reliably determine Ca(I)/Ca(II) preference or substitution formation energies in crystalline HAp, although they may provide qualitative information about local electronic descriptors.

### 5.4. From Site Preference to Structure–Property Relationships

The most robust computational studies distinguish clearly between three related but non-equivalent questions: whether incorporation of a dopant is thermodynamically favorable relative to chemically meaningful reservoirs; which combination of substitution site and compensating defect has the lowest energy; how an experimentally plausible defect ensemble modifies the properties of HAp?

Many publications address only the third question by constructing an assumed doped composition and calculating its band structure, density of states, optical response, or elastic constants. These calculations can reveal trends within a consistent family of models, but they do not establish that the selected configurations are thermodynamically accessible.

This distinction is especially important when interpreting band-gap narrowing. Introduction of transition-metal or rare-earth states near the band edges can reduce a Kohn–Sham gap, but a smaller calculated gap does not by itself demonstrate enhanced electrical conductivity. Conductivity also depends on defect ionization, carrier concentration, mobility, state localization, and polaron formation. Moreover, semilocal DFT systematically underestimates the band gaps of insulating materials, and open-shell dopants may require spin polarization, hybrid functionals, DFT+U, or more advanced treatment.

Three methodological developments offer a route toward more reliable predictions. First, machine-learning-assisted configurational search can identify low-energy dopant distributions that would be missed by a small set of manually generated models. Second, finite-temperature free-energy calculations can connect 0 K DFT energies to site occupancies established during synthesis. Third, direct calculation of spectroscopic observables-such as EPR, XPS, XAS, NMR, or vibrational parameters-provides a means of validating atomistic models against experiment.

The collective literature therefore supports a shift away from describing doped HAp through a single optimized structure (Table 1). A realistic computational representation should be viewed as an ensemble of substitutional and compensating defects whose populations depend on composition, chemical environment, synthesis temperature, hydration, and thermal history. Within this framework, Ca(I)/Ca(II) preference is not the final result of a calculation but one component of a broader defect-chemical and statistical-mechanical description.

## 6. Anionic Substitution in Hydroxyapatite

Anionic substitution in hydroxyapatite involves two chemically and structurally distinct sublattices: the tetrahedral phosphate framework and the one-dimensional hydroxyl channels extending parallel to the crystallographic *c*-axis. The former can accommodate oxyanions such as SiO_4_^4−^, CO_3_^2−^, and NO_3_^−^, whereas the latter is most commonly modified by monovalent anions such as F^−^. Because the substituting species may differ from the host anion in charge, geometry, protonation state, and coordination requirements, anionic incorporation is frequently coupled to hydroxyl vacancies, calcium vacancies, proton transfer, or other compensating defects.

As in the case of cationic substitution, a nominal chemical composition does not uniquely define the atomistic structure of an anion-modified HAp. The same substituent may be incorporated through several charge-neutral mechanisms, whose relative stability depends on water activity, oxygen and hydrogen chemical potentials, synthesis temperature, and local defect association. Moreover, bulk incorporation, adsorption at a surface, and non-equilibrium filling of pre-existing vacancies constitute fundamentally different processes and should not be discussed interchangeably.

### 6.1. Silicate Substitution and Charge Compensation

Silicon-modified hydroxyapatite is commonly described as arising from replacement of PO_4_^3−^ by SiO_4_^4−^. This notation is chemically incomplete because each isolated silicate-for-phosphate substitution introduces one additional negative charge. A realistic atomistic model must therefore specify how charge neutrality is restored.

Several possible mechanisms have been evaluated using first-principles calculations. Using effective defect notation relative to the perfect HAp lattice, the principal charge-compensation schemes can be represented as

$$Si_{P}^{\prime }+V_{OH}^{\cdot }$$

for silicate substitution compensated by a hydroxyl vacancy;

$${\left(HSiO_{4}\right)}_{P}^{\times }$$

for protonated silicate substitution; and

$$2Si_{P}^{\prime }+V_{O}^{\cdot \cdot }$$

for two silicate substitutions compensated by one oxygen vacancy. Here, ′ denotes one negative effective charge, · one positive effective charge, ·· two positive effective charges, and × an effectively neutral defect relative to the corresponding site in stoichiometric HAp. Configurations containing HPO_4_^2−^ or condensed Si_2_O_7_ units have also been considered [14].

Astala et al. [14] treated the principal Si-related compensation mechanisms as overall charge-neutral defect complexes rather than as isolated charged defects. These included $Si_{P}^{\prime }+V_{OH}^{\cdot }$, $2Si_{P}^{\prime }+V_{O}^{\cdot \cdot }$, and neutral protonated ${\left(HSiO_{4}\right)}_{P}^{\times }$ configurations. Because these models differ in H and O content, their absolute energies cannot be compared directly. The authors therefore connected them through hydration reactions and expressed their relative formation energies as functions of the water chemical potential. Pure HAp, an isolated H_2_O molecule, and ice Ic were calculated as reference systems. When formation-energy differences are taken, most elemental chemical-potential terms cancel, leaving $\mu \left(H_{2}O\right)$ as the principal variable. Under relatively dehydrating conditions, OH-vacancy compensation was favored, whereas at higher water chemical potential protonated HSiO_4_ became more stable. The oxygen-vacancy-compensated model remained slightly above the lowest-energy envelope. The reported value of approximately 0.2 eV is therefore not an absolute formation energy; it denotes the approximate lowering in energy per Si impurity that would be required for this mechanism to become competitive over a narrow intermediate range of $\mu \left(H_{2}O\right)$ [14].

The preferred defect configurations further demonstrate that charge-compensating species should not be positioned arbitrarily. For OH-vacancy compensation, silicate tetrahedra located in the same phosphate layer and close to the missing hydroxyl group were approximately 0.4 eV more stable than more distant configurations. The substitutional anion and its compensating vacancy therefore behave as an associated defect complex rather than as independent, randomly distributed point defects [14].

This association is consistent with a general electrostatic principle observed throughout substituted HAp: separation of oppositely charged effective defects can be energetically expensive. Consequently, models in which the compensating vacancy, proton, or cation deficiency is placed far from the substituted tetrahedron may substantially overestimate the energy of incorporation.

An independent study focused on the OH-vacancy mechanism and evaluated the replacement of P by Si at each of the phosphate sites in the HAp unit cell. Calculations were performed using CASTEP, a plane-wave basis, and ultrasoft pseudopotentials. One Si atom and one OH vacancy in the conventional unit cell corresponded to an effective silicon content of approximately 2.8 wt.%, which is relatively high compared with many experimentally prepared single-phase Si-HAp materials [33].

Chappell and Bristowe [33] employed a different thermodynamic construction. For the charge-compensated model, one Si-for-P substitution was accompanied by removal of one OH group, and the formation energy was referenced to OH-deficient HAp together with the chemical potentials of P and Si. Several possible P and Si reservoirs were examined; the selected lowest-energy combination used phosphorus oxide as the P sink and cristobalite as the Si source, giving a formation energy of approximately −0.61 eV. The authors then repeated the analysis for a Si-for-P model in which both hydroxyl groups remained present. Using the same Si and P chemical potentials, this model gave a formation energy of approximately +3.53 eV, corresponding to an upward shift of approximately 4.14 eV. Importantly, the latter model is chemically uncompensated in the formal defect-chemistry sense, but it was not treated as an explicitly charged q≠0 periodic supercell with a compensating electrostatic background. Thus, the 4.14 eV difference should not be interpreted as a charged-cell finite-size artifact. It reflects the energetic consequence of the different stoichiometric and charge-compensation models within the formation-energy definitions adopted in that study [33]. Accordingly, the ~0.2 eV value reported by Astala et al. and the ~4.14 eV difference reported by Chappell and Bristowe describe different quantities and should not be compared quantitatively.

A second Si substitution, accompanied by removal of the remaining hydroxyl group, was in most configurations almost as favorable as the first. In one arrangement, the second substitution was even more favorable, suggesting an attractive interaction or cooperative stabilization between neighboring silicate-related defects. However, the corresponding composition was approximately 5.6 wt.% Si, at which experimental materials may undergo phase transformation or formation of other calcium silicates and phosphates. Such transformations cannot emerge spontaneously from calculations constrained to retain the periodic HAp topology [33].

Silicate substitution generally expands the local tetrahedral environment because Si–O bonds are longer than P–O bonds and SiO_4_ occupies a larger volume than PO_4_. The resulting change in the unit-cell parameters nevertheless depends on the compensating defect. Protonation, hydroxyl removal, and oxygen vacancies affect the lattice differently and can explain why experimental studies have reported apparently inconsistent variations in *a*, *c*, and cell volume. Calculated expansion or contraction should therefore not be attributed to silicon incorporation alone without identifying the associated hydrogen and oxygen stoichiometry.

The predicted electronic effects also depend on concentration and structural model. In an OH-deficient unit-cell calculation, silicate substitution reduced the Kohn–Sham band gap by approximately 1.6 eV. The newly introduced states were located deep within the valence band rather than inside the band gap, indicating that Si incorporation did not intrinsically convert HAp into an electrically active material [33].

This result should be distinguished from the creation of shallow electronic defects or mobile charge carriers. A decrease in the calculated band gap does not necessarily imply increased conductivity, especially when the changes originate from occupied bonding states located well below the valence-band maximum.

### 6.2. Surface Structure and Dissolution of Silicate-Substituted HAp

The enhanced bioactivity and biodegradability frequently reported for Si-HAp have often been attributed directly to increased dissolution of the substituted crystal lattice. Periodic surface calculations, however, suggest that the relationship is more complex.

A later study used a 2 × 2 × 2 HAp supercell containing one silicate substitution and one hydroxyl vacancy, corresponding to approximately 0.35 wt.% Si. This lower concentration reduced artificial interactions between periodic defect images and was closer to the composition of many experimental single-phase materials. The most stable bulk arrangement placed SiO_4_^4−^ and the OH vacancy in the same unit cell and phosphate layer [15].

Slab models based on the optimized bulk structure showed similar surface energies for pristine HAp and Si-HAp(001), but their charge distributions differed significantly. In an implicit solvent environment, surface calcium ions in Si-HAp became more positively charged, while oxygen atoms belonging to SiO_4_ became more negative than phosphate oxygen atoms. These changes produced a modified electrostatic potential above the surface and could alter interactions with proteins, amino acids, cell-membrane components, and solvate [15].

By contrast, the calculated barriers for removing exposed calcium ions from HAp and Si-HAp surfaces were similar. The solvent reduced the energetic cost of separating Ca^2+^ from both surfaces, but silicate substitution did not produce a major additional reduction. The experimentally observed increase in Si-HAp dissolution may therefore arise predominantly from microstructural factors such as reduced grain size, increased specific surface area, grain boundaries, amorphous regions, and secondary phases rather than from an intrinsically weaker Ca binding energy in an ideal crystalline surface [15].

This distinction is important because an ideal, charge-neutral slab represents only one component of the experimental dissolution process. Real dissolution also involves sequential protonation of surface phosphate groups, explicit water coordination, calcium hydration, cooperative removal of several ions, pH-dependent surface charge, and continuous surface reconstruction. Pulling one ion away from a selected surface position provides a useful comparative descriptor but does not constitute a complete dissolution mechanism.

Silicon can therefore influence bioactivity through several routes that are not equivalent: modification of surface electrostatics, changes in morphology and grain size, increased defect density, altered protein adsorption, and potentially different phase compositions. Direct attribution of enhanced biological response to one calculated bulk or surface property should be avoided.

### 6.3. Fluoride Substitution and Surface Stabilization

Replacement of OH^−^ by F^−^ is formally isovalent and does not require additional charge compensation. Nevertheless, the distribution of fluoride between the surface and bulk and its ordering within hydroxyl channels remain important. The fluoride study discussed below is retained despite its substantial classical-molecular-dynamics component because it represents a multiscale framework rather than an independent empirical-force-field dataset. The HAp interatomic potential was parameterized against experimental structural and thermochemical data and subsequently validated against electronic-structure calculations for fluoride-containing HAp and hydroxyfluorapatite solid solutions [34]. The first-principles calculations therefore establish the energetic consistency of the bulk substitution model, whereas molecular dynamics extends the analysis to explicitly hydrated surfaces, fluoride uptake from solution, segregation, and dissolution processes that require substantially larger systems and longer time scales. Accordingly, the MD-derived results are used here as complementary mechanistic evidence and are distinguished from direct DFT results throughout the discussion.

DFT and DFT-validated interatomic-potential calculations showed that replacement of OH^−^ by F^−^ is thermodynamically favorable in bulk and mixed hydroxyfluorapatite models, whereas classical MD was used to examine the subsequent behavior of fluoride at the hydrated surface [34]. Mixed hydroxyfluorapatite compositions may exhibit non-random ordering, with a preference for planes enriched in F^−^ or OH^−^ perpendicular to the *c*-axis rather than simple alternation of the two species along individual hydroxyl channels [34].

The behavior of fluoride introduced from aqueous solution is strongly surface-selective. Molecular-dynamics simulations gave an incorporation energy of approximately −193 kJ mol^−1^ for substitution in the outermost layer. Incorporation into the second and third subsurface layers remained favorable but became progressively weaker, with values of approximately −164 and −68 kJ mol^−1^, respectively. Further migration beyond approximately 10 Å into the bulk was nearly thermoneutral, with an energy of only about −4 kJ mol^−1^ [34].

These results suggest that exposure of HAp to fluoride does not necessarily produce a uniformly fluorinated bulk crystal. Instead, it can generate a thin fluorapatite-like surface region. This surface layer inhibits the onset of dissolution through two complementary effects. First, substitutional fluoride remains bound within the lattice more strongly than the surface hydroxyl groups that may dissolve into water. Second, F^−^ coordinates surrounding calcium ions and helps anchor them to the apatite surface [34].

The simulations additionally highlight the importance of hydration in surface modelling. At a vacuum interface, extensive relaxation of the Ca and phosphate sublattices disrupts the pairing and orientation of hydroxyl groups, producing configurations that differ significantly from a hydrated surface. In the presence of water, coordination of surface calcium by solvent molecules reduces this reconstruction and largely preserves hydroxyl ordering. Vacuum-slab calculations may therefore provide a misleading representation of hydroxyl-channel structure when the intended application involves aqueous or biological environments.

The shorter communication devoted to fluoride-induced resistance to dissolution used the same hydrated HAp(0001) slab, classical potential model, simulation temperature, and principal incorporation energies [35]. It should therefore be regarded as an earlier presentation of the same modelling framework rather than as an independent confirmation of the extended study [34]. The latter provides the more complete account because it includes DFT validation of the potential model, solid-solution calculations, fluoride segregation, and detailed surface analysis.

The fluoride studies also illustrate the need to distinguish the methodological origin of individual conclusions. Bulk defect and solid-solution energetics were evaluated or validated using DFT, whereas fluoride uptake from water, diffusion, and dissolution resistance were examined primarily using classical molecular dynamics. These results remain relevant to anionic modification but should not be described collectively as direct DFT simulations.

### 6.4. Nitrate Impurities and Competition with Carbonate

Nitrate represents a less common but instructive example of anionic incorporation. It can be retained as a trace impurity during wet synthesis using nitrate-containing precursors and subsequently converted by ionizing radiation into the paramagnetic NO_3_^2−^ radical detectable by EPR.

Two possible substitution modes were considered: A-type substitution in the hydroxyl channel and B-type substitution at a phosphate site.

Because replacing PO_4_^3−^ with NO_3_^−^ changes the formal charge by two units, the B-type model was compensated by a neighboring calcium vacancy. The calculations employed Quantum ESPRESSO, PBE, an 88-atom monoclinic HAp cell, and full structural relaxation. Spin-polarized calculations were used for the irradiated NO_3_^2−^ radical, and GIPAW was employed to calculate the ^14^N hyperfine and *g* tensors [36].

Formation-energy calculations favored B-type occupation over insertion into the hydroxyl channel across a broad range of chemical conditions [36]. More importantly, the calculated spectroscopic parameters for the B-type NO_3_^2−^ radical agreed closely with experiment. The calculated principal components of the *g* tensor were approximately 2.0054, 2.0052, and 2.0021, compared with experimental values of about 2.0055, 2.0055, and 2.0015. The calculated hyperfine components were likewise close to the measured values, whereas the A-type model produced less satisfactory tensor magnitudes and orientations [36].

This represents a particularly strong use of first-principles modelling. The structural assignment is not based solely on relative total energies but is independently tested through a directly measurable spectroscopic observable. The close agreement between the periodic GIPAW model and EPR also suggests that, for crystallites of approximately 30 nm, the detected nitrate radicals are predominantly bulk-like rather than surface-localized.

Carbonate and nitrate can compete for related substitutional environments. Experimentally, increasing carbonation progressively decreased the nitrate-derived EPR signal. A calculated comparison of selected B-type defects favored carbonate incorporation over nitrate by approximately 5.53 eV [36]. This supports the interpretation that carbonate introduced during synthesis can displace or suppress nitrate impurities.

The numerical value should nevertheless be interpreted within its thermodynamic definition. The comparison used a particular carbonate configuration, Na-assisted charge-compensation scheme, and selected reservoirs for Na, C, and N. It should not be regarded as a universal carbonate–nitrate exchange energy valid under all synthesis conditions. The qualitative conclusion that carbonate is more readily incorporated is, however, supported independently by the experimental loss of nitrate-related EPR intensity.

The nitrate study also demonstrates that anions present only in trace quantities can be structurally important and may be invisible to conventional XRD, FTIR, or elemental analysis. Combining calculated EPR parameters with irradiation-generated radical probes provides an unusually sensitive means of identifying such impurities and their lattice positions.

### 6.5. Surface Incorporation of Silicon-Containing Species

Not every material described experimentally as “silicon-doped HAp” necessarily contains substitutional SiO_4_^4−^ at phosphate sites. During solution combustion synthesis, silicon-containing species may adsorb on growing calcium-phosphate surfaces, segregate at grain boundaries, or participate in secondary phases.

A combined experimental and computational study of solution-combustion-derived calcium phosphates modelled adsorption of Si–OH species on HAp(001). The calculated adsorption energy was approximately 1.53 eV, and the results were correlated with changes in surface roughness, crystal-growth direction, and oxygen-reduction activity [37].

This model describes surface association during crystal growth rather than replacement of PO_4_^3−^ by SiO_4_^4−^ in bulk HAp. It does not compare protonated silicate, hydroxyl vacancies, oxygen vacancies, or other charge-compensation schemes. Accordingly, it should not be used as evidence for the preferred bulk substitution mechanism.

The reported reduction in the oxygen-reduction barrier and its proposed relationship with antioxidant behavior should also be interpreted cautiously. A model surface reaction provides mechanistic insight into selected elementary steps, but cellular oxidative stress depends on a much broader network of reactions, solution conditions, surface terminations, and biological processes.

### 6.6. Hydroxyl Vacancies and Non-Equilibrium Oxygen Incorporation

Hydroxyl vacancies are not only charge-compensating defects but can also serve as sites for subsequent non-equilibrium anion incorporation. A recent high-performance plasma-doping approach exploited OH-deficient HAp produced by thermal treatment and exposed it to oxygen-containing non-thermal plasma.

Experimentally, plasma treatment introduced an additional oxygen species detected by XPS and improved the electrical response of HAp by up to approximately one order of magnitude. The maximum measured surface concentration of the assigned oxygen dopant was 5.62%. The authors proposed that ionized oxygen species initially occupy surface hydroxyl vacancies and subsequently migrate through the OH channels into the material [38].

To assess the feasibility of transport, DFT calculations were performed for 1 × 1 × 4 supercells containing 169 atoms. The model columns contained an intentionally high vacancy concentration of 50%, and sequences of oxygen and hydroxyl displacements were examined using nudged elastic band calculations. The calculated migration barriers were generally around 1 eV [38].

Electron-localization analysis suggested that the positively charged region associated with an OH vacancy attracts electron density belonging to the oxygen dopant toward the bulk. The authors proposed that energetic electrons and electrical fields generated under plasma conditions could assist in overcoming the calculated migration barriers. However, the plasma sheath, external electric field, ion-impact dynamics, and non-equilibrium electronic distribution were not explicitly included in the static DFT and NEB models [38].

The oxygen-modified HAp exhibited increased catalytic activity for CO_2_ conversion, with the best material showing an approximately 230% increase in total product yield relative to untreated HAp. The improvement was attributed to a combination of enhanced electrical properties and the possible role of incorporated oxygen species as binding or reactive sites [38].

These results are promising but should not be interpreted as a conventional equilibrium substitution of one lattice anion by another. The model represents a deliberately vacancy-rich, externally driven, non-equilibrium system. Moreover, the bulk oxygen concentration was inferred indirectly rather than quantified independently, and the calculated diffusion pathway depended on predefined rules for collective movement within the hydroxyl columns.

The study is therefore most appropriately discussed as an example of vacancy-assisted, non-equilibrium anion engineering, illustrating how the hydroxyl-channel architecture of HAp can be exploited for post-synthetic modification.

### 6.7. General Trends and Methodological Implications

The available computational literature on anion-modified HAp is smaller than that devoted to cation substitution, but it reveals several consistent principles.

First, heterovalent oxyanion substitution must be described together with charge compensation. Replacing PO_4_^3−^ by SiO_4_^4−^ or NO_3_^−^ without adding or removing other species creates a periodically charged or chemically unrealistic structure. Hydroxyl vacancies, protonation, calcium vacancies, oxygen vacancies, and coupled cation substitutions can lead to different geometries and properties even at the same nominal dopant concentration.

Second, the stable defect may depend on the surrounding chemical environment. For Si-HAp, dehydrating conditions favor an OH-vacancy mechanism, whereas hydration favors protonated HSiO_4_. A single zero-temperature structure therefore cannot represent the entire synthesis-dependent defect chemistry.

Third, bulk incorporation and surface uptake must be distinguished. Fluoride introduced from solution strongly stabilizes the outer surface but shows little thermodynamic driving force for deep penetration into the crystal. Silicon-containing species may adsorb on HAp during crystal growth without occupying phosphate positions in the bulk. Surface-sensitive experiments may therefore detect a dopant even when its lattice concentration is limited.

Fourth, atomistic conclusions should be matched to the method used. Static periodic DFT is well suited to comparing relaxed defect configurations and relative energies. Classical molecular dynamics can access hydrated surfaces and ion motion over hundreds of picoseconds but depends on the transferability of the potential model. Slab calculations with implicit solvent capture some dielectric stabilization but do not reproduce proton transfer and solvent restructuring explicitly. Non-equilibrium processes such as plasma doping require additional external fields and excited species that are not present in conventional ground-state calculations.

Fifth, direct calculation of spectroscopic parameters provides one of the strongest routes for validating anion-defect models. The assignment of nitrate to a phosphate site was supported not only by formation energies but also by quantitative agreement between calculated and experimental EPR tensors. Similar strategies using vibrational spectra, solid-state NMR, XPS core-level shifts, and X-ray absorption could greatly improve future structural assignments.

The limited number of studies also exposes important gaps. Carbonate is the most abundant substitutional anion in biological apatite, yet systematic first-principles comparisons of A-type, B-type, and mixed A/B carbonate configurations remain underrepresented. Likewise, relatively little attention has been given to chloride, bicarbonate, sulfate, and other oxyanions under consistent defect-formation formalisms.

Overall, anion-modified hydroxyapatite should be viewed as an ensemble of coupled substitutional, protonic, and vacancy defects whose populations depend on synthesis conditions and interfacial environment (Table 2). The term “anion-doped HAp” is insufficient unless the substituted site, compensating defect, protonation state, and distinction between bulk and surface incorporation are explicitly defined.

## 7. Co-Doping and Coupled Substitutions in Hydroxyapatite

Co-doping extends the chemical flexibility of hydroxyapatite beyond the effects achievable through an isolated substitution. Two or more foreign species may occupy equivalent calcium sites, different calcium sublattices, or distinct cationic and anionic sites. In heterovalent systems, one substituent may additionally compensate the effective charge introduced by another. Consequently, the atomistic entity controlling stability and properties may be a spatially associated defect complex rather than either dopant considered independently.

The term co-doping nevertheless encompasses several fundamentally different situations. A negative interaction or binding energy provides evidence of genuine energetic synergy between two defects. Structural compensation occurs when one dopant reduces the lattice distortion caused by another, even when a discrete bound complex cannot be identified. Functional complementarity refers to systems in which different dopants independently provide desirable properties, such as osteogenic and antibacterial activity. Finally, many reported co-doped HAp models contain two elements in an assumed periodic composition but do not test their relative positions, charge compensation, or mutual interaction. These categories should not be regarded as equivalent.

A useful energetic measure of defect association is

$$\Delta E_{int}=E_{HAp+A+B}+E_{HAp}-E_{HAp+A}-E_{HAp+B},$$

where all terms are evaluated using cells of the same size, structural convention, and computational settings. A negative value indicates that simultaneous incorporation is more favorable than formation of the two isolated defects. This approach has been applied explicitly only in a limited number of HAp studies, most convincingly for Mn^2+^/NO_3_^−^ and Mg^2+^/CO_3_^2−^ defect pairs.

### 7.1. Coupled Cation–Anion Substitutions

#### 7.1.1. Association Between Manganese and Nitrate Defects

The simultaneous incorporation of Mn^2+^ and nitrate provides one of the clearest demonstrations that the preferred position of a cation can be modified by a pre-existing anionic defect. The study combined pulsed EPR with periodic PBE+U calculations performed using Quantum ESPRESSO. An 88-atom monoclinic HAp cell was used, with selected results checked in a 176-atom model. A Hubbard parameter of 3 eV, determined through a linear-response procedure, was applied to the localized Mn 3d states [39].

For isolated Mn^2+^ substitution, Ca(II) was calculated to be slightly more favorable than Ca(I) (Figure 3). The energy difference between the most stable Ca(II) position and the examined Ca(I) sites was approximately 0.06–0.13 eV. Mn incorporation shortened the local Mn–O distances relative to the corresponding Ca–O contacts, reflecting the smaller ionic radius of Mn^2+^ [39].

The nitrate impurity was represented as a B-type defect replacing PO_4_^3−^, with charge compensation provided by a neighboring calcium vacancy. The position of the vacancy was first optimized for nitrate-containing HAp and subsequently kept fixed while different Mn positions were tested. Pair-binding energies became increasingly negative as Mn approached the nitrate–vacancy complex. The strongest association, approximately −0.30 eV, was obtained for Mn at the nearest Ca(I) site, with an Mn–N separation of about 0.34 nm. Mn located at a neighboring Ca(II) site gave a weaker stabilization of approximately −0.15 eV at 0.63 nm, whereas more distant configurations were stabilized by only about −0.05 eV [39].

This result is particularly important because the Ca(I) configuration favored in the presence of nitrate was not the preferred position of isolated Mn. The anionic defect therefore reshaped the local site-preference landscape. The authors attributed this association primarily to compensation of local lattice distortions rather than to direct electrostatic or magnetic attraction, because both isolated model defects were formally neutral and the spin density of Mn^2+^ remained strongly localized. The geometrically complementary distortions produced by the two impurities lowered the energy of the combined system [39].

Pulsed EPR relaxation measurements independently indicated that Mn^2+^ influenced the relaxation of radiation-generated NO_3_^2−^ centers. The experimental results were consistent with a non-random Mn distribution around nitrate impurities, although direct Mn–nitrate distances could not be extracted using the attempted ENDOR or DEER measurements. Thus, the existence of an interaction was supported experimentally, while the precise associated geometry was derived primarily from DFT [39].

The Mn/nitrate system establishes a general principle for multi-substituted HAp. The site preference of a dopant cannot necessarily be transferred from the pristine lattice to a material containing other substitutional or vacancy defects. Trace impurities inherited from synthesis precursors may therefore influence the uptake, spatial distribution, and subsequent release of intentionally introduced therapeutic ions.

#### 7.1.2. Mutual Incorporation of Magnesium and Carbonate

The Mg^2+^/CO_3_^2−^ system represents an even more direct example of local cation–anion complex formation. Calculations were performed in an 88-atom monoclinic HAp cell using Quantum ESPRESSO, the PW91 functional, ultrasoft pseudopotentials, a 45 Ry wave-function cutoff, and a 2 × 2 × 1 *k*-point mesh. Both atomic coordinates and cell dimensions were relaxed [40].

Two carbonate environments were examined. In A-type substitution, CO_3_^2−^ occupies the hydroxyl channel and replaces channel anions. In B-type substitution, carbonate replaces PO_4_^3−^ and requires additional charge compensation. For the latter, one Ca^2+^ ion was replaced by Na^+^, reflecting the frequent coexistence of sodium and carbonate in biological apatite. Mg^2+^ was placed at both Ca(I) and Ca(II) sites and at different distances from carbonate.

The most favorable interaction was obtained for A-type carbonate combined with Mg^2+^ at a neighboring Ca(II) site. The calculated interaction energy was −0.308 eV, and the Mg–O distance decreased to 1.98 Å. This contact was shorter than the Mg–O distances involving phosphate or hydroxyl oxygen atoms. By contrast, Mg occupying a remote Ca(I) position could not interact directly with the channel carbonate, and its interaction energy was close to zero [40].

For B-type carbonate, the predicted interaction with Mg was much weaker. Depending on the locations of Mg and the compensating Na ion, the calculated stabilization ranged from approximately −0.037 to −0.076 eV. The Mg–carbonate distances were longer, and no similarly compact complex was formed [40].

The calculations therefore suggest that Mg can selectively stabilize A-type carbonate. This result was consistent with previously reported FTIR changes indicating an increased relative contribution of A-type carbonate in Mg-containing HAp. The computational model provides a plausible atomistic explanation: Mg at Ca(II) surrounds the anionic channel and can coordinate carbonate directly, whereas Ca(I) is structurally separated from the channel [40].

The model does not imply that all Mg-containing biological apatite should contain predominantly A-type carbonate. Carbonate distributions also depend on synthesis conditions, temperature, hydration, calcium deficiency, and additional ions. Moreover, B-type models included Na^+^ and were therefore effectively three-defect systems. Nevertheless, the study demonstrates that the experimentally observed correlation between two impurities may arise from formation of a specific short-range complex rather than merely from their common presence in the lattice.

#### 7.1.3. Strontium and Fluoride Co-Substitution

Sr^2+^/F^−^ co-doping illustrates both structural compensation and functional complementarity. Sr^2+^ is larger than Ca^2+^ and generally expands the HAp lattice, while replacement of OH^−^ by the smaller F^−^ ion contracts and orders the hydroxyl channel. A combined experimental and DFT investigation examined 18 models containing Sr, F, or both dopants. Calculations were carried out using CASTEP/PBE, a plane-wave cutoff of 380 eV, a 4 × 4 × 4 *k*-point mesh, and unconstrained relaxation of lattice parameters and atomic positions [41].

For 10 mol.% Sr, Ca(II) substitution was favored over Ca(I) by approximately 0.283 eV. At 20 mol.% Sr, the lowest-energy configuration instead placed two Sr ions at Ca(I), although the difference between the highest- and lowest-energy configurations was only 0.066 eV and should therefore be regarded as a weak energetic ordering rather than a uniquely resolved configuration. The result again demonstrates that site occupancy may change with concentration and the presence of multiple dopants [41].

F substitution produced negative formation energies and became increasingly favorable as the hydroxyl channel approached the fluorapatite composition. All Sr/F models had more negative formation energies than the corresponding Sr-only structures. F incorporation also reduced the expansion of Sr–O distances and partly suppressed the lattice deformation caused by Sr. The authors therefore concluded that fluoride stabilizes Sr-substituted HAp [41].

Unlike the Mn/NO_3_ and Mg/CO_3_ studies, however, no Sr–F pair-binding energy was defined. The more negative energy of a codoped composition may partly reflect the intrinsically favorable replacement of OH^−^ by F^−^ rather than a specific attraction between Sr and F. The study consequently supports global structural stabilization but does not prove formation of a discrete Sr–F complex.

The biological functions of the dopants were also largely complementary. Fluoride-containing samples displayed antibacterial activity against *Streptococcus mutans*, whereas an appropriate Sr concentration promoted osteogenic differentiation. Codoped materials combined these effects without introducing measurable cytotoxicity under the tested conditions [41].

### 7.2. Cation–Cation Co-Doping

#### 7.2.1. Magnesium and Zinc

Mg^2+^ and Zn^2+^ are both smaller than Ca^2+^ and contract the HAp lattice, but their preferred calcium environments differ. In a combined experimental and DFT study, isolated Mg showed a weak preference for Ca(I), with an energy difference of approximately 0.060 eV, whereas Zn preferred Ca(II) by approximately 0.048 eV. When both ions were introduced into the same unit cell, however, the lowest calculated formation energy corresponded to a configuration in which both occupied Ca(I) sites [42].

This result illustrates that independently determined site preferences are not necessarily additive. Nevertheless, all calculated formation energies were positive, and the study did not calculate an explicit Mg–Zn binding energy. The lower energy of one codoped configuration therefore identifies the preferred arrangement among the tested models but does not by itself demonstrate favorable co-incorporation relative to separate single-dopant structures.

Both Mg and Zn reduced the lattice parameters and crystallinity. At moderate experimental concentrations, nominally 5% Mg and 5% Zn, HAp remained the principal phase and exhibited improved cell response and antibacterial properties. At 10% Mg and 10% Zn, however, the material transformed into a whitlockite-like phase rather than remaining a highly substituted HAp solid solution [42].

This phase transformation is an important warning for periodic modelling. A calculation constrained to the HAp topology can produce a relaxed local minimum even when the real material lowers its free energy through transformation to another calcium-phosphate phase. Predictions made for highly substituted HAp should therefore be compared with experimental phase analysis before they are interpreted as properties of a stable HAp solid solution.

A separate theoretical study of Mg addition to Zn-based HAp reported progressive band-gap narrowing with increasing Mg concentration [43]. However, it focused on electronic structures of assumed compositions and did not evaluate the relative formation energies of competing Mg/Zn distributions or alternative phases. Its results are therefore more informative regarding model-dependent electronic trends than regarding the thermodynamics of co-doping.

#### 7.2.2. Strontium and Zinc

Sr^2+^ and Zn^2+^ provide a structurally complementary pair because Sr expands the lattice whereas Zn contracts it. Calculations comparing four possible Sr/Zn arrangements found Ca(II) to be preferred for both isolated dopants, by approximately 0.283 eV for Sr and 0.081 eV for Zn. Among the codoped models, the arrangement with both dopants at Ca(II) was the lowest in energy, while the complete spread between the tested configurations was approximately 0.257 eV [44].

The opposing effects of Sr and Zn reduced the net change in average lattice parameters relative to the respective singly substituted materials. Experimentally, the codoped scaffolds combined the osteogenic influence associated with Sr and the antibacterial activity associated with Zn. However, Zn-containing samples also showed secondary Zn-bearing calcium-phosphate phases, indicating that not all nominal Zn necessarily occupied substitutional HAp sites [44].

All calculated substitution formation energies were positive. As in the Mg/Zn work, no interaction energy was calculated relative to independently doped structures. The apparent structural compensation between a large and a small ion should therefore be distinguished from thermodynamic pair association.

The Sr/Zn system is a useful example of how co-doping may minimize macroscopic lattice changes while still producing substantial local distortions. A nearly unchanged average unit-cell volume does not imply that Sr and Zn occupy random or undistorted environments; local Sr–O expansion and Zn–O contraction may coexist and partly cancel in the crystallographic average.

### 7.3. Rare-Earth and Heterovalent Cation Pairs

A substantial fraction of computationally assisted co-doping studies concerns pairs of rare-earth elements or combinations of rare-earth and other heavy-metal ions. Examples include Ce/Sm [45], Ce/Yb [46], Pr/Ce [47], Y/Er [48], Pr/Er [49], Sr/Sm [50], La/Mg [51], Bi/Ce [52], and Sn/Bi [53].

These publications commonly combine wet-chemical synthesis with XRD, vibrational spectroscopy, thermal analysis, cell-viability measurements, and periodic band-structure calculations. The experimental portions provide useful information on lattice parameters, crystallinity, phase purity, and biological response. The computational sections, however, often represent a more limited category of modelling than the defect-complex studies discussed above.

Typically, one dopant concentration is held constant while the second is increased. A periodic cell corresponding to each nominal composition is then optimized, followed by calculation of the density of states and band gap. For example, the calculated gap of Ce/Sm-HAp decreased from 4.6078 to 4.0477 eV with increasing Sm content [45]. Pr addition to Ce-based HAp reduced the reported gap from 4.5156 to 4.3097 eV [47]. Pr/Er-HAp showed a smaller decrease from approximately 4.17 to 4.06 eV [49], whereas La/Mg-HAp was reported to decrease from approximately 4.58 to 4.44 eV [51].

The central difficulty is charge compensation. Trivalent Ce^3+^, Sm^3+^, Y^3+^, Er^3+^, Pr^3+^, La^3+^, Bi^3+^, and Yb^3+^ cannot all replace Ca^2+^ without additional defects. Possible mechanisms include calcium vacancies, hydroxyl deprotonation, anionic substitutions, and mixed oxidation states. Sn^4+^ substitution introduces an even larger excess positive charge. Unless these compensating species are explicitly included, the calculated electronic structure corresponds to an idealized or electronically compensated periodic model rather than to a chemically defined neutral defect ensemble.

This limitation is particularly consequential for band-gap calculations. Artificially added or removed electrons, delocalized background compensation, or an unspecified oxidation state may create states near the band edges. Apparent gap narrowing can then originate from the construction of the model rather than from a physically realistic defect complex.

Open-shell rare-earth ions introduce an additional challenge. Spin-unpolarized calculations or semilocal functionals without an appropriate description of localized 4f electrons may position the dopant-derived states incorrectly. A monotonic band-gap trend should therefore not automatically be interpreted as improved electrical conductivity, optical activity, or photocatalytic performance.

Experimental phase analysis also indicates that complete substitution should not be assumed. In Ce/Sm-HAp, detected dopant concentrations were lower than the nominal values, implying partial incorporation [45]. Pr/Ce samples contained a minor β-TCP phase whose abundance increased with Pr content [47]. Bi/Ce-HAp likewise contained β-TCP in addition to the main apatite phase [52]. In Pr/Er-HAp, experimental observations suggested that part of the dopant could remain at the surface rather than enter the lattice.

These studies remain valuable for identifying composition-dependent experimental trends, but their computational contribution should be described as electronic-structure modelling of assumed co-doped compositions, not as a first-principles determination of co-doping mechanisms.

### 7.4. Methodological Requirements for Predictive Co-Doping Models

Reliable modelling of multi-substituted HAp requires a larger configurational space than single-dopant calculations. For two dopants A and B, the model must consider Ca(I)/Ca(II), phosphate, and hydroxyl-channel site combinations; multiple A–B separations and relative orientations; alternative vacancy or protonation configurations; possible clustering and segregation; competing secondary phases and different oxidation or spin states.

The number of configurations grows rapidly with concentration. Evaluating only one codoped structure can therefore be less reliable than evaluating several single-dopant positions. Machine-learning-assisted configurational search, cluster expansion, or systematic enumeration could substantially improve future studies.

Charge compensation must be specified explicitly. A nominal formula containing heterovalent ions is not an atomistic defect model. The compensating calcium vacancy, hydroxyl vacancy, proton, substituted anion, or electronic charge should be included and its position optimized. For pairs such as Pr^3+^/Ag^+^, the two substitutions can in principle provide mutual charge compensation when they replace two Ca^2+^ ions in an appropriate ratio. By contrast, Pr^3+^/Zn^2+^ does not form a charge-neutral pair and requires an additional defect. Comparing their electronic structures without accounting for this difference can be misleading.

Formation and binding energies should also use chemically meaningful reference states. Total energies of cells with different compositions cannot be compared directly. Absolute defect formation energies require consistent elemental or compound reservoirs and, for charged defects, treatment of the Fermi level and finite-size corrections. Pair-binding energies avoid some reservoir dependence because the compositions cancel, but all component cells must use identical size and computational settings.

Finite-temperature effects remain almost entirely unexplored for co-doped HAp. Configurational entropy may favor a distribution of associated and unassociated defects rather than one ordered ground state. Vibrational contributions, hydration, and synthesis-dependent chemical potentials may further change the populations of competing complexes.

The experimental composition used for validation should be measured rather than inferred solely from precursor ratios. Partial incorporation, surface enrichment, amorphous material, β-TCP, whitlockite, and dopant-containing oxide or phosphate phases are common at higher concentrations. Agreement between a calculated lattice parameter and powder XRD does not uniquely establish that all nominal dopants occupy the modelled lattice positions.

Finally, direct simulation of observables offers the strongest route to structural validation. EPR relaxation and hyperfine parameters were particularly useful for Mn/NO_3_. Comparable validation could be achieved using solid-state NMR, XANES/EXAFS, XPS core-level shifts, vibrational frequencies, and pair-distribution functions.

### 7.5. General Conclusions

The co-doping literature demonstrates that the properties of multi-substituted HAp cannot generally be predicted by simply adding the effects of isolated dopants. An anionic impurity can reverse a cation’s preferred calcium site, as observed for Mn in nitrate-containing HAp. Direct Mg–carbonate coordination can selectively stabilize A-type carbonate. Fluoride can counteract Sr-induced lattice expansion, while oppositely sized Sr and Zn ions can reduce net changes in average lattice dimensions.

At the same time, relatively few studies calculate the interaction between dopants explicitly. Much of the current literature demonstrates either structural compensation or functional complementarity rather than thermodynamic synergy. Rare-earth co-doping studies often provide valuable experimental characterization but employ computational models with predefined dopant positions, incomplete charge compensation, and limited treatment of localized electronic states.

Co-doping becomes more than the simultaneous presence of two elements. It represents a controlled modification of defect chemistry in which local association, long-range distribution, synthesis conditions, and competing phases jointly determine the resulting structure and functionality (Table 3, Figure 4).

## 8. Structural and Thermodynamic Stability

The structural response of hydroxyapatite to ionic substitution is governed by more than the difference between the ionic radii of the host and substituting species. Larger divalent ions, such as Sr^2+^, Cd^2+^, and Pb^2+^, generally expand the lattice, whereas smaller ions, including Mg^2+^ and Zn^2+^, tend to shorten local metal–oxygen distances and reduce the unit-cell volume. Nevertheless, changes in the crystallographic parameters represent averages over the entire structure and may conceal substantial local distortions. A nearly unchanged unit-cell volume can therefore coexist with strongly contracted dopant–oxygen polyhedra, rotated phosphate groups, displaced hydroxyl ions, or opposing distortions generated by two different dopants.

The response also depends on the occupied sublattice. Ca(I) is coordinated exclusively by phosphate oxygen atoms, whereas Ca(II) is located adjacent to the hydroxyl channel. Consequently, substitution at Ca(II) can couple directly to hydroxyl displacement, deprotonation, channel contraction, or anionic substitution. This distinction explains why site preferences cannot be predicted reliably from ionic radius alone. Zn^2+^ and Mg^2+^, for example, have both been reported at different Ca sites depending on composition, channel chemistry, and the number and distribution of dopants [11,12,19].

Aliovalent substitution introduces a further structural variable because the compensating defect may control the lattice response more strongly than the dopant itself. Hydroxyl deprotonation and calcium-vacancy compensation produced different Eu distributions and different changes in lattice parameters, despite identical nominal Eu concentrations [7]. Similarly, SiO_4_^4−^ substitution can be coupled to an OH vacancy or protonation of the silicate tetrahedron, with the preferred mechanism depending on water chemical potential [14].

Thermodynamic conclusions require careful distinction between relative configuration energies, substitution energies, defect formation energies, and mixing energies. Relative energies of models with identical composition can be compared directly. By contrast, formation energies for different compositions depend on the selected chemical reservoirs and cannot be compared across studies unless the underlying reactions and chemical potentials are equivalent.

The importance of the chemical environment is illustrated particularly well by Fe-HAp. Under Ca-poor conditions, substitutional Fe at calcium sites was favored, whereas Ca-rich conditions stabilized interstitial Fe near the hydroxyl channel [6]. Thus, the preferred defect is not an intrinsic property of the element alone but a conditional result defined by the synthesis environment.

The calculated stability of a doped HAp configuration also does not establish the stability of HAp against competing phases. At high substitution levels, the experimental material may form β-TCP, whitlockite, CaO, dopant oxides, metal phosphates, or amorphous calcium phosphate. This limitation was apparent for highly Mg/Zn-substituted samples, where the HAp topology used in the calculations was replaced experimentally by a whitlockite-like phase [42]. Periodic optimization constrained to HAp can identify a local minimum even when another phase is thermodynamically preferred.

Solubility limits should therefore be evaluated through a combination of defect formation energies, mixing enthalpies, configurational entropy, and stability against competing phases. The few available finite-temperature studies indicate that entropy can change conclusions derived at 0 K. In Sr-HAp, a slight energetic preference for Ca(I) was replaced by mixed Ca(I)/Ca(II) occupation under experimentally relevant temperatures [13]. Overall, substituted HAp should be treated as a synthesis-dependent defect ensemble rather than as one perfectly ordered ground-state structure.

## 9. Electronic, Optical, Magnetic, and Mechanical Properties

The electronic structure of stoichiometric HAp is dominated by oxygen states at the top of the valence band and calcium-derived states in the conduction region. Semilocal DFT functionals generally underestimate its wide band gap, and absolute values should therefore not be compared directly between calculations employing LDA, PBE, PBEsol, hybrid functionals, or different pseudopotentials. Relative trends are more meaningful when the same computational protocol is applied consistently.

Isovalent substitution can modify the band edges through local bonding changes without necessarily creating states within the gap. Si substitution, for example, altered the valence-band structure and reduced the calculated Kohn–Sham gap in some models, but the additional states remained deep in the valence band rather than generating mobile carriers [33]. A smaller calculated gap should therefore not automatically be interpreted as enhanced electrical conductivity or photocatalytic activity.

Intrinsic vacancies and transition-metal dopants can instead introduce localized defect levels. Oxygen-, hydrogen-, and hydroxyl-related vacancies have been predicted to produce different in-gap states and changes in the work function [5]. The energetic position and occupation of such states depend on defect charge, local geometry, and the functional used. Hybrid DFT, DFT+U, and spin-polarized calculations are therefore particularly important for Fe-, Mn-, Co-, Ni-, and Cu-containing HAp.

Magnetic behavior is primarily associated with partially filled d or f shells. Mn substitution was predicted to introduce spin-polarized states and concentration-dependent magnetization, while Fe defects exhibited different magnetic moments depending on their substitutional or interstitial character and charge state [6,24]. Spin polarization must be included explicitly, and several oxidation and spin states should be considered before assigning the experimentally observed magnetism to a particular defect.

A substantial group of studies has reported band-gap narrowing in rare-earth-co-doped HAp. Such calculations are useful for comparing the electronic structures of the assumed periodic compositions, but their interpretation is limited when 4f electrons, oxidation states, and charge compensation are not treated explicitly. Changes in calculated band gaps should not alone be presented as evidence of improved luminescence, conductivity, radiation shielding, or photocatalytic efficiency.

Mechanical properties are affected by both global lattice changes and local strain. Mg has been predicted to increase selected elastic moduli in some models, whereas Zn frequently reduces stiffness or strength at higher concentrations [16]. Sr generally expands and slightly softens the lattice, although the outcome depends on concentration and occupied Ca sites. Co-doping with ions of opposite size can reduce changes in the average lattice constants, but this does not eliminate local strain around either ion.

Elastic constants calculated for one ordered periodic configuration describe an ideal single crystal at 0 K. Experimental hardness, fracture toughness, and compressive strength additionally depend on porosity, grain size, crystallinity, secondary phases, interfaces, and specimen preparation. Agreement between a calculated elastic modulus and a macroscopic experimental property should therefore be interpreted cautiously.

Protonic and ionic transport remain comparatively underexplored. Hydroxyl channels may support proton, hydroxyl, fluoride, or oxygen-related migration, but activation barriers alone do not determine conductivity. The concentration of mobile defects, their charge state, association with dopants, and temperature-dependent dynamics must also be considered. Static calculations should therefore be complemented by nudged elastic band calculations, phonons, and ab initio molecular dynamics.

## 10. Surfaces, Hydration, and Interfacial Interactions

The biological and catalytic performance of HAp is governed primarily by its surface rather than by the ideal bulk crystal. Relevant surface models must specify the crystallographic orientation, termination, stoichiometry, slab thickness, surface charge, and hydration state. Low-index surfaces such as (001), (010), and basal surfaces perpendicular to the hydroxyl channels have been examined most frequently, but direct comparison between studies is difficult because different terminations may expose Ca-rich, phosphate-rich, or hydroxyl-rich environments.

Doping can affect surface behavior even when it has only a minor effect on the bulk lattice parameters. Si-substituted HAp was predicted to exhibit a modified electrostatic potential, with more negatively charged silicate oxygen atoms and more positive exposed calcium ions. However, the calculated energy required to remove surface Ca remained similar to that of undoped HAp [15]. This suggests that the increased dissolution often observed for experimental Si-HAp may arise partly from smaller crystallites, grain boundaries, amorphous regions, and greater surface area rather than from an intrinsically weaker ideal surface.

Hydration can substantially alter surface reconstruction. In vacuum, undercoordinated calcium and phosphate groups may undergo displacements that are suppressed when water restores part of their coordination environment. Implicit-solvent models capture dielectric screening but cannot reproduce individual hydrogen bonds, proton transfer, or competitive adsorption. Explicit water is therefore preferable for dissolution, ion exchange, and biomolecular adsorption, although meaningful results require configurational sampling or molecular dynamics.

Fluoride provides a clear example of the distinction between surface uptake and bulk substitution. Simulations predicted strongly favorable incorporation in the first surface layers but little driving force for deep penetration into the crystal. Fluoride can consequently form a thin fluorapatite-like surface region that anchors calcium ions and increases resistance to dissolution without producing a uniformly fluorinated bulk material [34].

Computational studies of HAp interfaces have also examined interactions with graphene oxide, polycaprolactone, organic modifiers, drugs, and adsorbed ions. These models provide useful atomistic information on coordination, hydrogen bonding, charge transfer, and preferred adsorption geometries. However, an adsorption energy for one molecular orientation on one ideal surface cannot represent the complete biological response. Protein adsorption, bacterial adhesion, osteoblast activity, and cytotoxicity depend on surface coverage, competitive adsorption, pH, ionic strength, dissolution products, nanoparticle morphology, and time-dependent cellular processes.

Accordingly, correlations between DFT and biological properties should be framed mechanistically rather than causally. A calculation may show that Zn or Ag substitution strengthens polymer adhesion or changes surface charge, but it cannot by itself demonstrate improved osseointegration or antibacterial performance. The strongest studies combine surface calculations with experimentally measured adsorption, ion release, spectroscopy, dissolution, or cell response while retaining a clear distinction between atomistic descriptors and biological endpoints (Figure 5).

## 11. Comparison with Experimental Results

First-principles calculations are most valuable when they predict or reproduce an experimentally measurable observable rather than only optimize an assumed defect model.

Changes in lattice parameters measured by XRD provide a useful initial check, particularly for large or small isovalent dopants. However, average cell expansion or contraction rarely identifies a unique lattice site. Similar changes can result from different combinations of dopants, vacancies, protonation states, residual strain, and secondary phases. Rietveld-refined occupancies are more informative but become uncertain at low dopant contents or when the substituent and Ca have similar scattering factors.

Vibrational spectroscopy is sensitive to local phosphate, hydroxyl, carbonate, and silicate environments. Calculated IR and Raman frequencies can support assignments of OH loss, HPO_4_ formation, carbonate type, or local distortion. Nevertheless, broad bands in nanocrystalline materials may contain contributions from surfaces, adsorbed water, amorphous phases, and several overlapping defect configurations.

XPS can constrain oxidation states and near-surface composition, but calculated core-level shifts must account for final-state screening, surface termination, and charging. XANES and EXAFS are especially valuable for determining local coordination numbers and metal–oxygen distances, although several structural models may reproduce similar first-shell signals.

EPR provides one of the strongest examples of direct atomistic validation. For nitrate-containing HAp, the calculated g and hyperfine tensors of the B-type nitrate model agreed closely with experiment and discriminated it from the competing hydroxyl-channel structure [36]. Similarly, crystal-field calculations can support assignments of Mn to Ca-related environments [25].

Solid-state NMR remains underused despite its ability to probe phosphate protonation, hydroxyl environments, carbonate substitution, structural water, and local dopant-induced disorder. Calculated chemical shifts, quadrupolar parameters, and J couplings could provide particularly sensitive tests of charge-compensation models.

Mechanical measurements, dissolution rates, and ion release are more difficult to compare directly with perfect-crystal calculations. Experimental results depend strongly on porosity, particle size, grain boundaries, phase purity, surface area, pH, and specimen processing. DFT can identify a plausible elementary mechanism or comparative trend, but it rarely predicts the full macroscopic response without complementary mesoscale or kinetic modelling.

Thus, agreement between a calculated property and one experimental trend supports a model but seldom proves it uniquely. The most convincing structural assignments arise when energetics, local geometry, and several independent observables converge on the same defect mechanism (Figure 6).

## 12. Current Limitations and Future Perspectives

The principal limitation of the existing literature is the absence of a standardized thermodynamic framework. Energies labelled as substitution, incorporation, or formation energies are often based on different reservoirs and sign conventions. Future studies should report the complete defect reaction, chemical potentials, competing phases, model composition, and reference states. Relative energies should be distinguished clearly from absolute formation energies.

A second limitation is the widespread use of small unit cells. A single substitution in a 44-atom HAp cell represents a high dopant concentration and enforces artificial periodic ordering. Larger supercells are required to describe experimentally relevant dilute compositions, isolated defects, and long-range relaxation. Convergence with respect to cell size should become a routine part of defect calculations.

Configurational sampling is equally important. For more than one dopant or compensating defect, numerous arrangements may differ by substantial energies. The Zn-HAp study combining DFT with a convolutional neural network demonstrated that limited or random placement can miss the low-energy region of configuration space [11]. Future work should employ symmetry-based enumeration, stochastic searches, cluster expansion, machine-learning potentials, or active-learning strategies.

Most calculations remain restricted to ordered structures at 0 K. Configurational entropy, vibrational free energies, hydration, and thermal expansion may change site occupancies and defect populations. Phonon calculations should be used to verify dynamical stability, while ab initio molecular dynamics can address hydroxyl reorientation, proton transfer, hydration, and finite-temperature coordination changes.

Water and surfaces also require greater attention. Biological HAp is nanocrystalline, hydrated, carbonated, calcium-deficient, and continuously interacting with ions and biomolecules. Perfect bulk stoichiometric HAp should therefore be treated as a reference model rather than a complete representation of bone mineral. Explicitly hydrated surfaces, grain boundaries, amorphous–crystalline interfaces, and realistic solution compositions are important future targets.

For transition-metal and rare-earth systems, spin polarization, alternative oxidation states, DFT+U or hybrid functionals, and relativistic effects should be applied systematically. Calculated band-gap narrowing should be supported by analysis of defect-state localization, carrier formation, and optical transition probabilities rather than used alone to infer conductivity or photocatalysis.

High-throughput DFT and machine learning offer a route to screening large numbers of dopants and configurations, but their usefulness will depend on the quality of the underlying defect models. Models trained on structures with missing charge compensation or unrealistic concentrations will reproduce those assumptions efficiently rather than correct them. ML predictions should therefore be followed by first-principles verification and experimental validation.

Finally, closer integration between computation and advanced characterization is essential. DFT calculations of solid-state NMR parameters, EPR tensors, XPS shifts, vibrational spectra, and XANES/EXAFS signatures can transform modelling from a post hoc interpretative tool into a method for discriminating competing defect structures. Microscopy and spatially resolved spectroscopy can further distinguish lattice incorporation from surface segregation and secondary-phase formation.

The most promising future framework is therefore not the optimization of one nominally doped structure, but the construction of a condition-dependent defect ensemble, taking into account composition and synthesis conditions, possible defects and complexes, finite-temperature populations, bulk and surface structures as well as calculation and comparison with measurable properties.

Figure 7 should be interpreted as a qualitative synthesis linking representative dopants with property changes and potential application domains, rather than as a map of mechanisms established directly by first-principles calculations. Some labels, including ROS generation and Fenton-like activity, represent downstream mechanistic concepts relevant to antibacterial or redox-active modified HAp systems. These processes are not directly demonstrated by the DFT calculations reviewed here and depend additionally on ion release, oxidation state, solution chemistry, surface reactivity, and the biological or catalytic environment. First-principles calculations can provide supporting atomistic descriptors, such as defect states, oxidation-state stability, adsorption energetics, or surface interactions, but experimental validation is required before such descriptors can be connected causally to ROS production, Fenton-like chemistry, or biological performance. Such an approach would allow computational modelling to move from qualitative rationalization toward predictive design of hydroxyapatites with controlled structural, biological, optical, magnetic, and catalytic functions (Figure 7).

## 13. Conclusions

First-principles calculations have substantially improved the atomistic understanding of ion-substituted hydroxyapatites by providing access to dopant locations, charge-compensation mechanisms, local distortions, defect association, and structure–property relationships that are often difficult to resolve experimentally. The available evidence demonstrates, however, that substitution-site preference is not a fixed property of an element. It depends on dopant concentration, supercell size, hydroxyl ordering, oxidation and spin states, compensating defects, chemical potentials, hydration, temperature, and the presence of other impurities or secondary phases.

The most reliable studies compare multiple configurations of chemically neutral defects, define formation energies using meaningful reference states, and validate the resulting models through directly measurable observables. In contrast, calculations performed for a single assumed composition may provide useful electronic or mechanical trends but do not necessarily establish the thermodynamic mechanism of incorporation. This distinction is particularly important for aliovalent and co-doped systems, in which the relevant structural entity is often an associated defect complex rather than an isolated substitution.

Future modelling should therefore describe modified HAp as a condition-dependent ensemble of bulk, surface, substitutional, protonic, and vacancy defects. Combining large-scale configurational sampling, finite-temperature calculations, explicit hydration, machine-learning-assisted searches, and first-principles spectroscopy offers the most promising route from qualitative interpretation toward the predictive design of hydroxyapatites with controlled biological and functional properties.

## Figures and Tables

**Figure 1:**
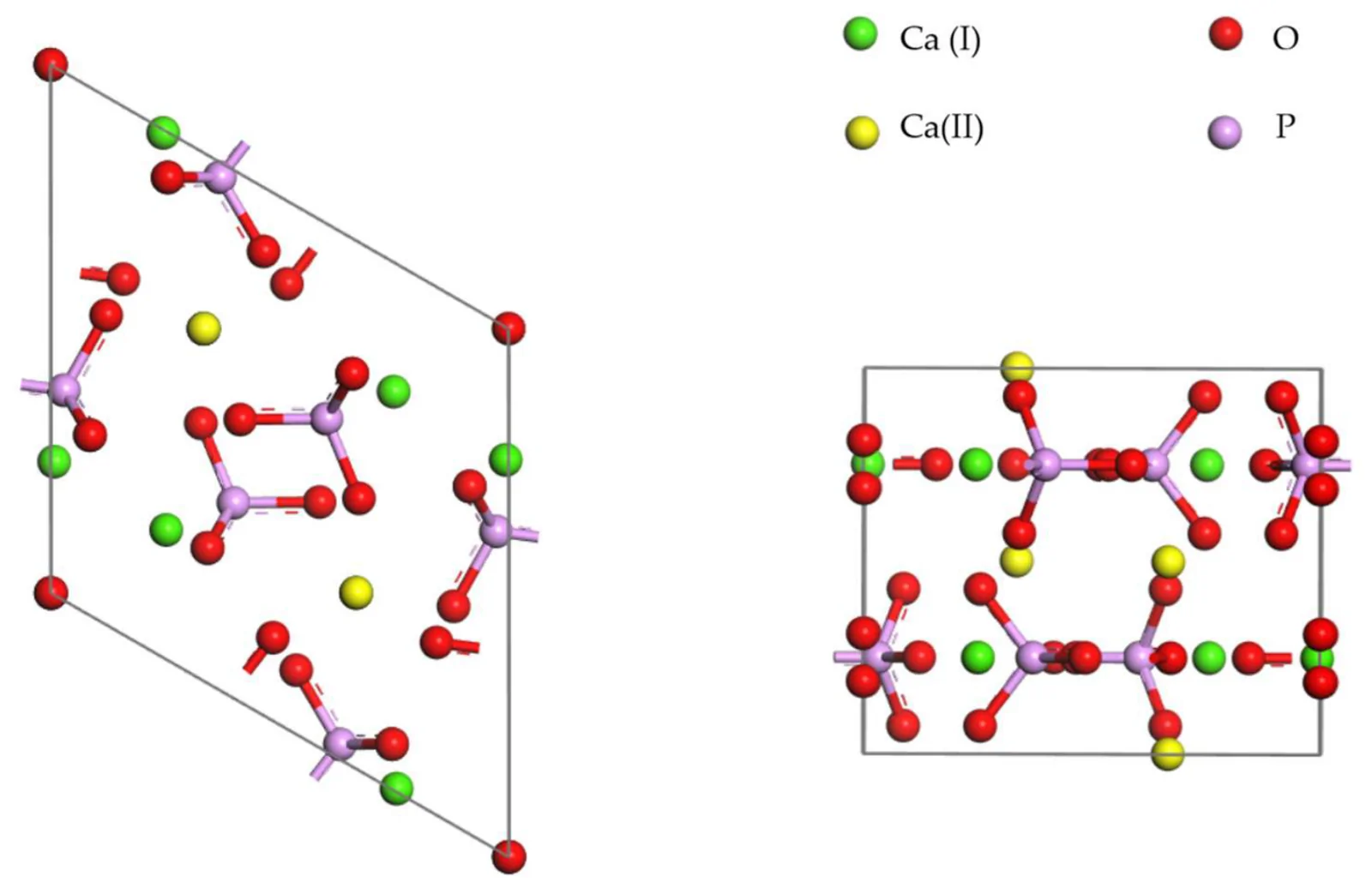
Crystal structure of HAp, refcode 2300273 from Crystallography Open Database https://www.crystallography.net/cod/search.php (accessed on 1 August 2026).

**Figure 2:**
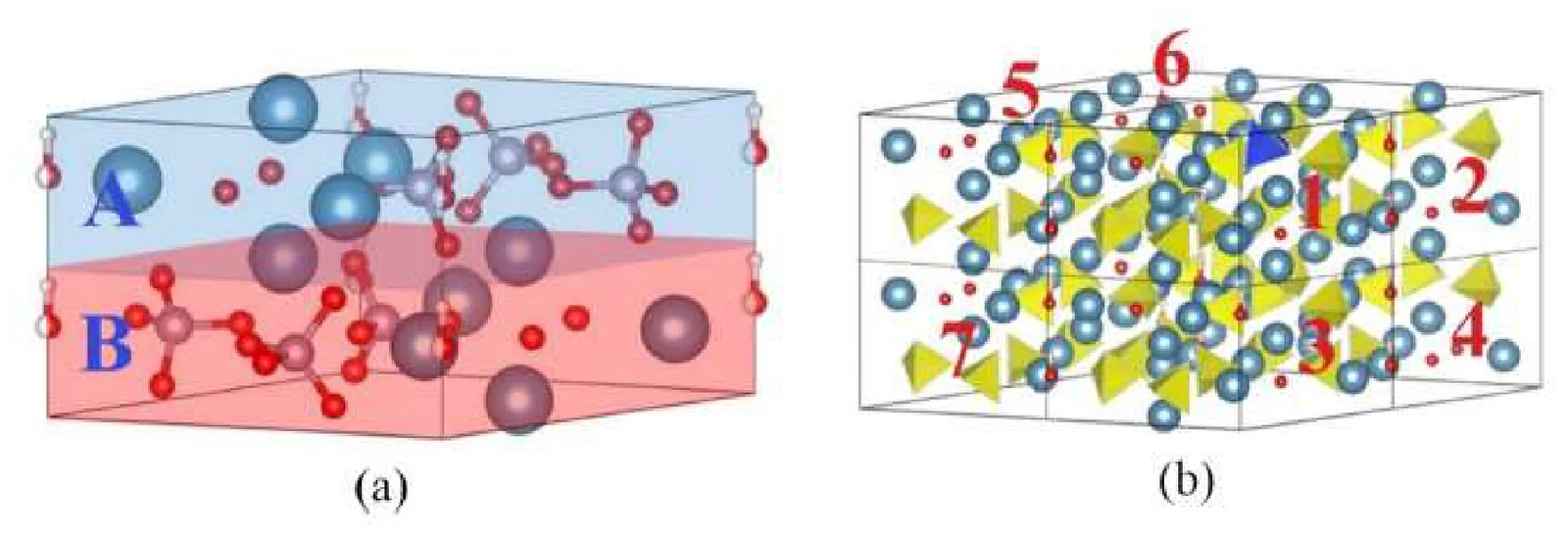
(**a**) Unit cell of HA. It has two layers, upper and lower. (**b**) Si-HA supercell with eight labeled unit cells. Reproduced, with permission, from [15].

**Figure 3:**
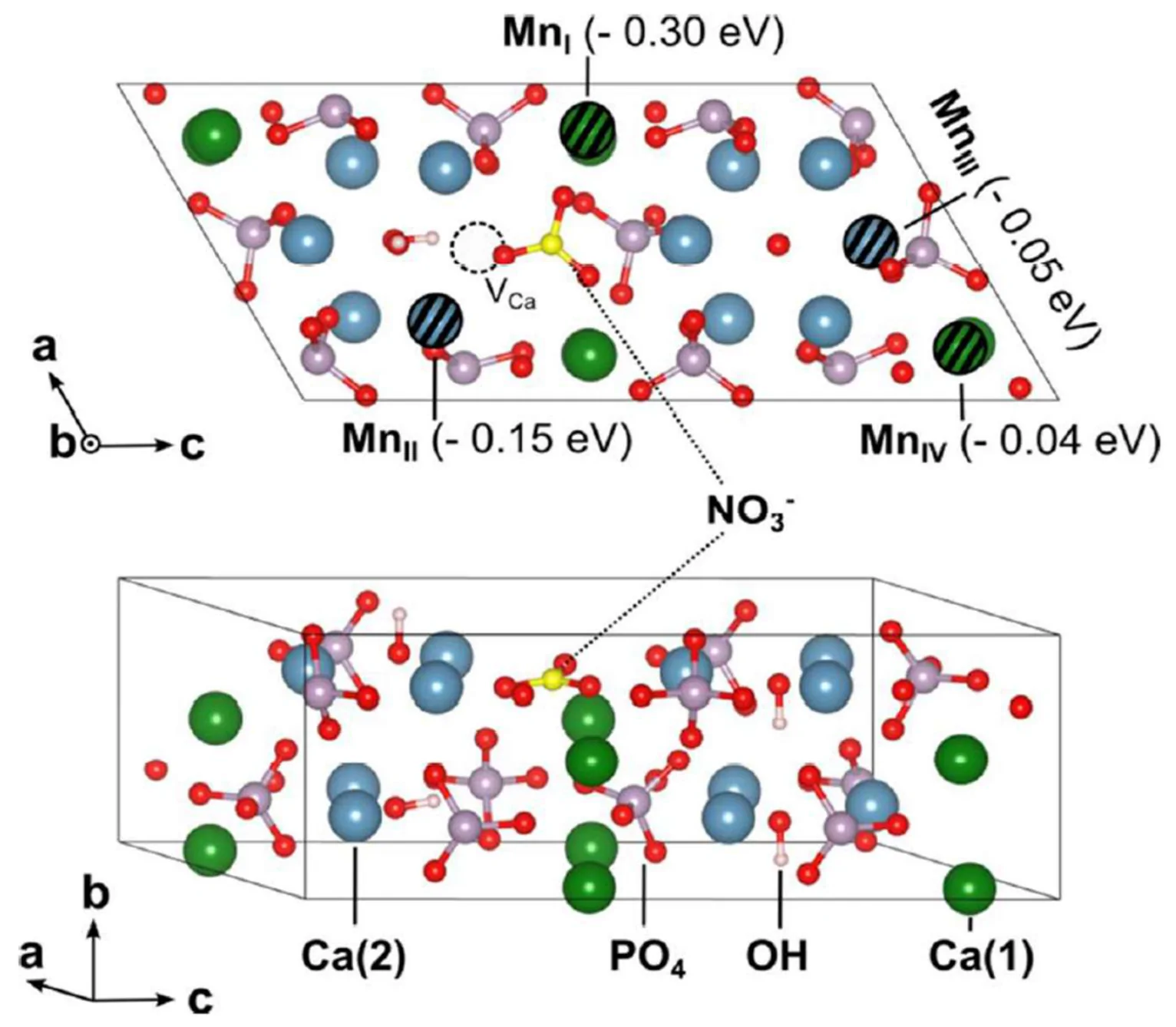
Optimized geometry of HAp containing NO_3_^−^ defect with schematic illustration of the selected positions of Mn^2+^ considered in the work. The values of binding energy calculated for the displayed Mn^2+^ sites are shown in parentheses (MnI and MnII represent the most stable configurations among Ca(1) and Ca(2) positions, respectively). Location of Ca^2+^ vacancy (VCa) is marked by a dashed circle. Ca(1), Ca(2), P, O, H and N atoms are shown as green, blue, purple, red, white and green spheres, respectively.

**Figure 4:**
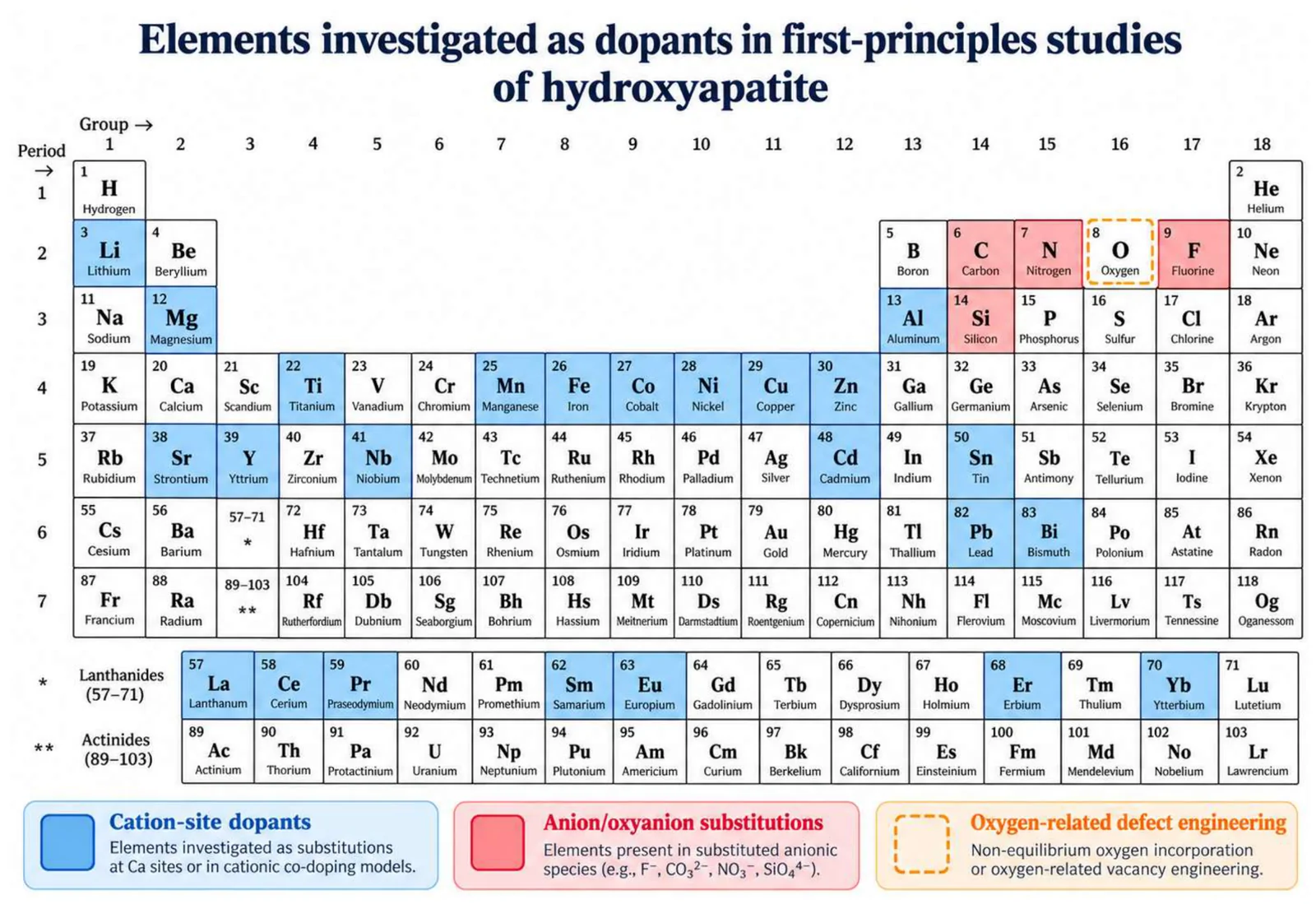
Periodic-table map of elements investigated in first-principles studies of ion-modified HAp included in the present review. Cation-site dopants are shown in blue, elements involved in anion/oxyanion substitutions in red, and oxygen-related defect engineering is indicated separately. The figure was generated with the assistance of ChatGPT version 5.6 Sol and subsequently manually verified and corrected by the authors.

**Figure 5:**
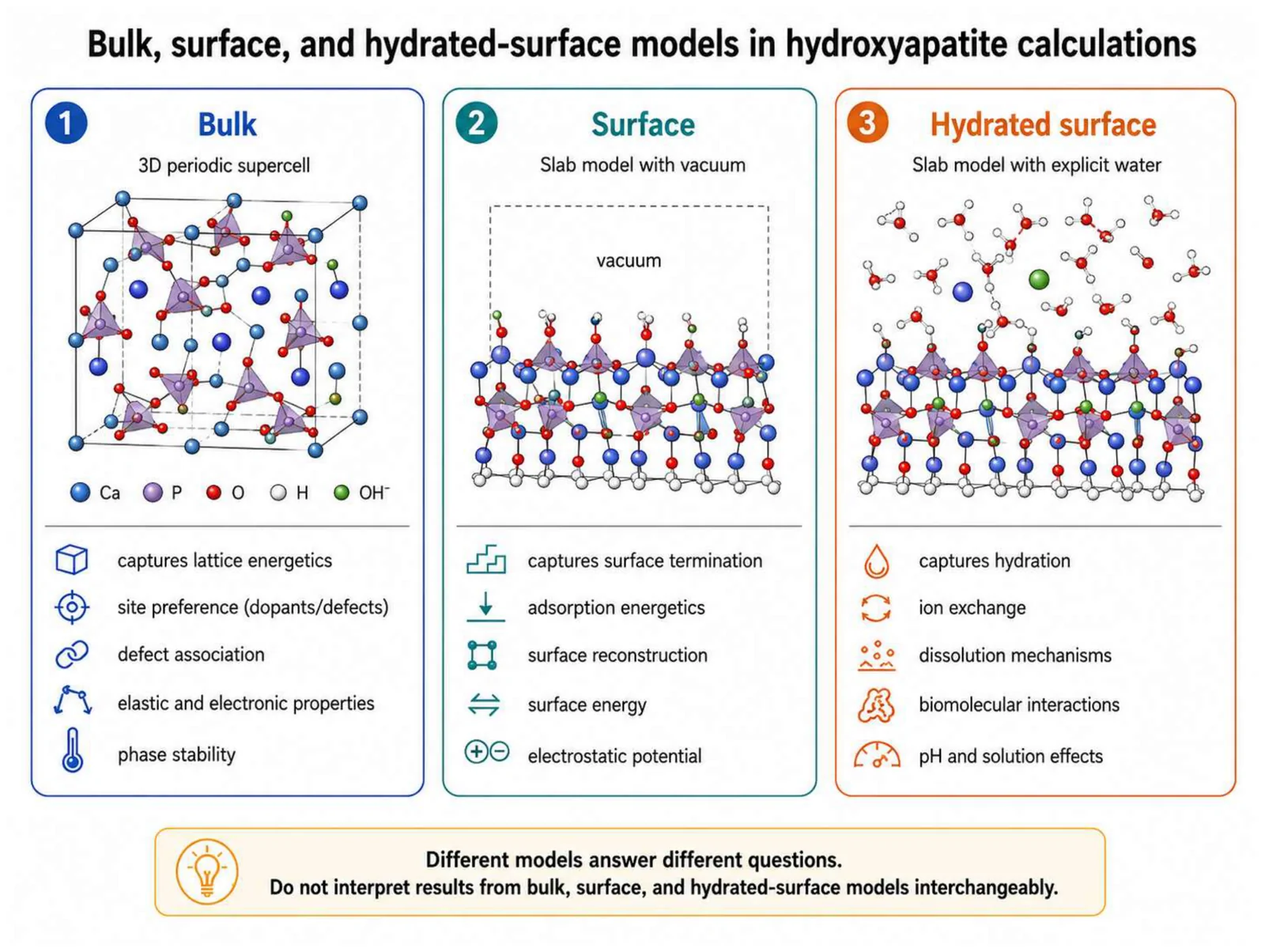
Bulk, surface and hydrated surface first-principles studies of HAp. Figure created using ChatGPT version 5.6 Sol.

**Figure 6:**
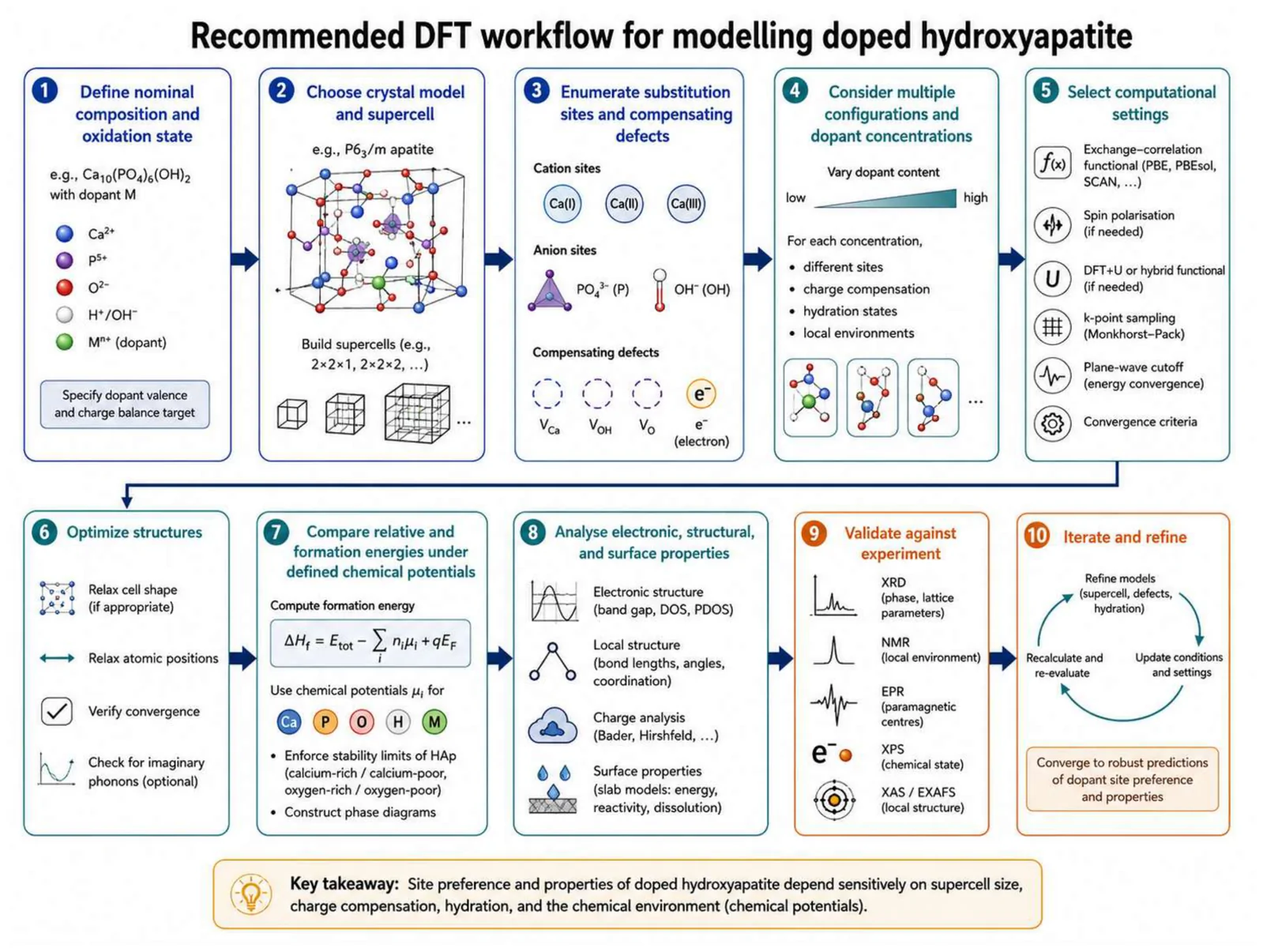
Recommended DFT workflow for modelling doped HAp. Figure created using ChatGPT version 5.6 Sol.

**Figure 7:**
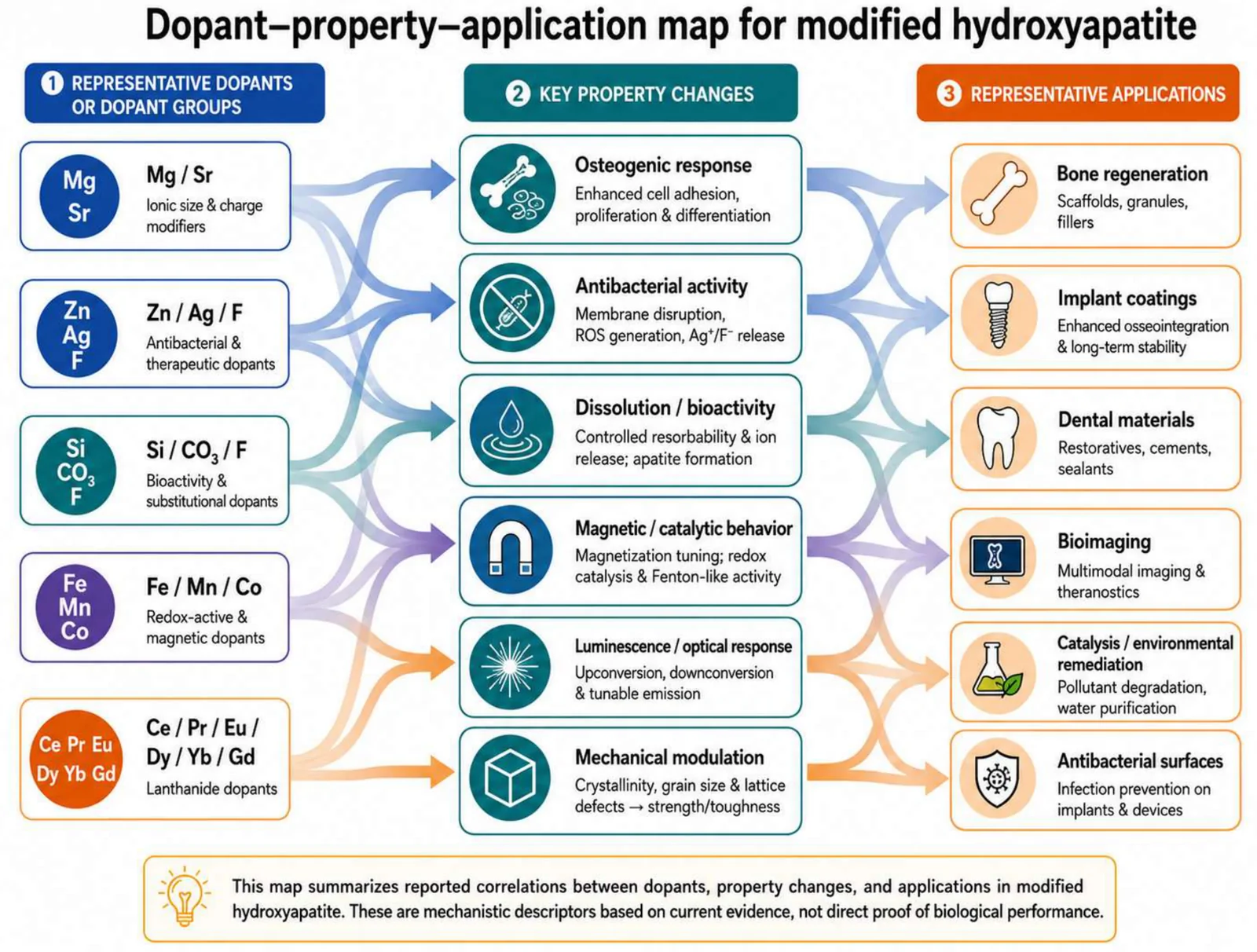
Dopant–property–application map for modified HAp. The diagram is intended as a qualitative synthesis; application-level mechanisms and outcomes are not necessarily established directly by first-principles calculations and require experimental validation. Figure created using ChatGPT version 5.6 Sol and scientifically verified by the authors.

**Table 1:** Representative DFT studies of cation-substituted hydroxyapatite.

Dopant(s)	Model/Composition	Preferred Site	Charge Compensation	Defect Charge Treatment	Method	Main Conclusion	Experimental Validation	Ref.
Mg^2+^	5 and 10% Mg substitution; conventional and 1 × 1 × 2 periodic cells	Slight preference for Ca(I)	Not required; isovalent Ca^2+^ to Mg^2+^ substitution	Neutral isovalent substitution; no charged-defect correction required.	CASTEP; periodic GGA-PBE DFT; full cell relaxation	Ca(I) was favored by only 0.012 and 0.077 eV at 5 and 10% Mg, respectively, indicating that both Ca sites may be accessible. Mg caused strong local contraction of the oxygen coordination environment.	XRD, XRF, electron microscopy, and thermal analysis supported limited Mg incorporation and lattice contraction; experimentally accessible substitution was approximately 5–7 mol.%.	[12]
Zn^2+^	2 × 2 × 2 supercell with 80 Ca sites; 1.25–6.25 mol.% Zn; thousands of configurations screened	Ca(II)	Not required; isovalent substitution	Neutral isovalent substitution; no charged-defect correction required.	VASP; GGA-PBE DFT combined with a convolutional neural network	Zn preferentially occupied Ca(II), but the relative positions of multiple Zn ions were also decisive. Symmetric distribution among Ca(II) channels minimized Zn–Zn interactions and lattice deformation; configurations with the same composition differed by more than 20 kcal mol^−1^.	No direct experimental validation; low-energy CNN-selected configurations were recalculated by DFT.	[11]
Sr^2+^	Multiple ordered Sr/Ca configurations over a broad solid-solution range; free energies evaluated at 90, 700, and 950 °C	Slight Ca(I) preference at 0 K; mixed Ca(I)/Ca(II) distribution at finite temperature	Not required; isovalent substitution	Neutral isovalent substitution; no charged-defect correction required.	Periodic DFT combined with an ideal configurational-entropy model	Although 0 K energies weakly favored Ca(I), configurational entropy stabilized mixed occupation under experimentally relevant synthesis conditions. Several mixed configurations differed by only a few kJ mol^−1^.	Rietveld refinement showed concentration-dependent and mixed Sr occupation, supporting a thermally averaged site distribution.	[13]
Cd^2+^	88-atom periodic models with several Cd concentrations and configurations; embedded clusters derived from optimized structures	Ca(II), particularly at moderate and high Cd contents	Not required; isovalent substitution	Neutral isovalent substitution; no charged-defect correction required.	Plane-wave periodic DFT and embedded-cluster electronic-structure analysis	Ca(II) preference was attributed not only to ionic size but also to stronger Cd–O and Cd–OH interactions. The preference became clearer as Cd concentration increased.	Synchrotron XRD and Rietveld analysis showed no resolvable preference at 1.2 at.% Cd but clear Ca(II) enrichment at 13 and 25 at.%.	[22]
Pb^2+^	Periodic unit-cell and embedded-cluster models spanning low to high Pb substitution	Ca(II) up to approximately 40% Pb; mixed Ca(I)/Ca(II) at 50–60%	Not required; isovalent substitution	Neutral isovalent substitution; no charged-defect correction required.	VASP; GGA plane-wave DFT; embedded clusters	Site preference changed with composition. Ca(II)-dominated models were favored at low and intermediate substitution, whereas mixed occupation became slightly more stable at high Pb content.	XRD, FTIR, and EXAFS supported preferential Pb occupation of Ca(II) at lower concentrations.	[23]
Mn^2+^	2 × 2 × 2 supercells containing different numbers and distributions of Mn	Ca(II) in the calculated concentrated periodic models	Not required for Mn^2+^ to Ca^2+^	Neutral isovalent substitution; no charged-defect correction required.	Quantum ESPRESSO; spin-polarized GGA-PBE geometry optimization followed by HSE/HSE06 hybrid-functional calculations; no DFT+U	Mn substitution contracted the lattice, introduced spin-polarized Mn-derived states within the HAp gap, and increased magnetization with dopant concentration.	Mechanochemical synthesis, XRD, and magnetic measurements supported Mn incorporation and magnetic response.	[24]
Fe^2+^ and Fe^3+^	88-atom monoclinic cell; Fe placed at Ca(I) and Ca(II)	Ca(II); weak preference for Fe^2+^ and stronger preference for Fe^3+^	Fe^2+^: none; Fe^3+^: OH deprotonation in the principal model	Fe^2+^: neutral isovalent substitution; Fe^3+^: neutral compensated complex via OH deprotonation.	Quantum ESPRESSO; periodic GGA-PBE DFT; no Hubbard U correction and no hybrid functional.	Fe^2+^ favored Ca(II) by approximately 0.2 eV, whereas deprotonation-compensated Fe^3+^ favored Ca(II) by approximately 1.03 eV. Fe^3+^ produced greater contraction and shorter Fe–O distances.	Primarily theoretical; consistent with spectroscopic assignments of Fe at Ca-related environments in related experimental studies.	[26]
Li^+^	88-atom monoclinic cell; Li at Ca(I) or Ca(II) with two alternative compensation mechanisms	Ca(I), by approximately 0.2 eV	Removal of OH^−^ or protonation of PO_4_^3−^ to HPO_4_^2−^	Neutral compensated complexes via OH removal or phosphate protonation.	Quantum ESPRESSO; periodic GGA-PBE DFT	Ca(I) substitution caused less lattice and hydroxyl-channel distortion. Both compensation routes favored Ca(I), while experimental observations supported compensation involving hydroxyl loss.	XRD, FTIR, and EPR; hydrogen-related centers were interpreted as evidence for OH-removal compensation.	[8]
Al^3+^	2 × 1 × 1 supercell; eight Al–Ca-vacancy configurations	Al at Ca(II), associated with a neighboring Ca(II) vacancy	Two Al^3+^ substitutions compensated by one Ca vacancy	Neutral compensated complex: 2Al^3+^ + one Ca vacancy.	VASP; periodic GGA-PBE DFT	The most stable structure contained an Al–vacancy complex involving Ca(II) sites. Relaxation displaced Al, rotated PO_4_ tetrahedra, and disturbed the hydroxyl channel.	XRD, FTIR, and microscopy supported Al incorporation and increasing structural disorder.	[29]
Eu^3+^	2 × 2 × 2, 352-atom models with several Eu arrangements	Moderate Ca(II) preference; both sites potentially accessible	OH deprotonation or Ca vacancy	Neutral compensated complexes via OH deprotonation or Ca vacancy.	Quantum ESPRESSO; periodic PBEsol DFT	OH deprotonation was more favorable than Ca-vacancy compensation and promoted a more homogeneous Eu distribution. Ca vacancies favored locally clustered Eu–vacancy structures. Ca(II) preference was modest rather than exclusive.	Theoretical study; designed to resolve competing charge-compensation models.	[7]
Ti^4+^ and Ti(OH)_2_^2+^	One Ti-containing species in a 1 × 1 × 1 HAp cell (~10 at.%); additional 1 × 1 × 2, 5 at.% check for Ti(OH)_2_^2+^.	Nearly no preference for uncompensated Ti^4+^; strong Ca(II) preference after compensation	Bare Ti^4+^: none; compensated Ti^4+^: removal of two channel protons; Ti(OH)_2_^2+^: intrinsically charge balanced relative to Ca^2+^.	Bare Ti^4+^→Ca^2+^: formally +2 effective defect, chemically uncompensated; no explicit charged-supercell correction reported. Ti^4+^ + removal of 2H^+^: neutral compensated complex. Ti(OH)_2_^2+^→Ca^2+^: neutral isovalent substitution.	VASP; periodic GGA-PW91 DFT	Site preference depended on the chemical identity of the incorporated species. Deprotonation made Ca(II) more stable by approximately 2 eV, while Ti(OH)_2_^2+^ formed a particularly stable Ca(II)-centered complex.	No direct experimental validation in the computational study; high model concentration was explicitly recognized as a limitation.	[9]
Co^2+^, Ni^2+^, Cu^2+^	44-atom HAp unit cell; one Ca replaced by Co, Ni, or Cu; Ca(I) and Ca(II) tested	Ca(II) among the tested models	Not required for nominal M^2+^ → Ca^2+^ substitution	Neutral isovalent substitution; no charged-defect correction required	ABINIT; LDA and GGA calculations; no DFT+U and no hybrid functional	Ca(II) was lower in energy for all three dopants in the tested unit-cell models; transition-metal-derived states modified the calculated band gap. LDA reproduced experimental gaps better than GGA within the adopted modelling scheme.	Hydrothermal synthesis; XRD, Raman, FTIR, TEM, and optical band-gap measurements	[27]

Abbreviations: DFT, density functional theory; DOS, density of states; EPR, electron paramagnetic resonance; GGA, generalized gradient approximation; HAp, hydroxyapatite; MD, molecular dynamics; NEB, nudged elastic band; PAW, projector augmented wave; PBE, Perdew–Burke–Ernzerhof functional; PW91, Perdew–Wang 1991 functional; XRD, X-ray diffraction.

**Table 2:** Representative DFT studies of anionic substitution and coupled defects in hydroxyapatite.

Dopant(s)	Model/Composition	Preferred Site	Charge Compensation	Defect Charge Treatment	Method	Main Conclusion	Experimental Validation	Ref.
SiO_4_^4−^	SiO_4_^4−^ replacing PO_4_^3−^; multiple protonic and vacancy-related defect models	PO_4_^3−^ site	OH vacancy under dehydrating conditions; HSiO_4_^3−^ formation under water-rich conditions; oxygen-vacancy and HPO_4_ models also tested	Neutral compensated defect complexes; OH-vacancy, protonated-silicate, and oxygen-vacancy schemes were constructed to maintain overall charge neutrality.	Periodic DFT using localized-orbital methods and chemical-potential-dependent formation energies	The preferred defect was controlled by water chemical potential. SiO_4_–OH-vacancy complexes were favored under dry conditions, whereas protonated HSiO_4_ became stable under hydrated conditions. Closely associated Si and vacancy positions were about 0.4 eV more stable than separated defects.	Theoretical study; the alternative mechanisms provided an explanation for synthesis-dependent experimental compositions.	[14]
SiO_4_^4−^	One or two Si substitutions in the conventional HAp cell; approximately 2.8 and 5.6 wt.% Si	PO_4_^3−^ site	One OH vacancy for each SiO_4_^4−^ substitution	Neutral OH-vacancy-compensated model; chemically uncompensated Si-for-P model also examined without an explicit charged-defect correction.	CASTEP; periodic plane-wave DFT	Removing OH^−^ reduced the calculated formation energy by approximately 4.14 eV relative to uncompensated substitution. A second Si substitution was sometimes nearly as favorable as the first, although the model concentrations approached the range where competing phases may form.	No direct experimental component; results were discussed in relation to experimentally accessible Si contents.	[33]
SiO_4_^4−^	2 × 2 × 2 bulk cell with one SiO_4_ substitution and one OH vacancy; HAp(001) slab in implicit solvent	PO_4_^3−^ site; Si and OH vacancy favored in the same layer	OH vacancy	Neutral compensated SiO_4_/OH-vacancy complex.	VASP; GGA-PBE/PAW; bulk and surface calculations with implicit solvation	Si substitution changed surface charge distribution and electrostatic potential but did not markedly lower the barrier for removing surface Ca. Higher experimental dissolution may therefore arise mainly from morphology, grain boundaries, and disorder.	Comparative interpretation against experimental reports of enhanced Si-HAp dissolution; no direct synthesis in the computational study.	[15]
F^−^	Hydroxyfluorapatite bulk configurations and hydrated HAp(0001) surface models	OH^−^ channel site; strongest incorporation at the outer surface layers	Not required; isovalent OH^−^ → F^−^ substitution	Neutral isovalent OH^−^ → F^−^ substitution; no charged-defect correction required.	DFT and interatomic-potential calculations combined with classical MD using METADISE and DL_POLY	F incorporation was strongly favorable at the surface but weakened with penetration depth: approximately −193, −164, and −68 kJ mol^−1^ for the first three layers and nearly thermoneutral deeper in the crystal. Fluoridation was predicted to form a thin, dissolution-resistant fluorapatite-like surface layer.	Computational study of hydrated surfaces; consistent with experimentally established fluoride-induced reduction in apatite dissolution.	[34]
NO_3_^−^/NO_3_^2−^	Nitrate in the OH channel or replacing PO_4_^3−^; irradiated NO_3_^2−^ radical used for spectroscopic assignment	B-type substitution at a PO_4_^3−^ site	Neighboring Ca vacancy	Neutral B-type nitrate + Ca-vacancy complex for structural energetics; spin-polarized radical model used for EPR assignment.	Quantum ESPRESSO; spin-polarized GGA-PBE DFT; GIPAW calculation of *g* and hyperfine tensors	Formation energies favored B-type nitrate. Calculated EPR tensors for the PO_4_-site model closely matched experiment, whereas the OH-channel model was less consistent. Carbonate was substantially more stable than nitrate in the selected defect scheme.	EPR, XRD, and electron microscopy; quantitative agreement between calculated and experimental EPR parameters provided direct structural validation.	[36]
Surface Si–OH species	Si-containing group adsorbed on HAp(001) during solution-combustion synthesis	Surface adsorption site rather than a demonstrated bulk PO_4_ substitution	Not formulated as a bulk charge-compensation mechanism	No isolated charged-defect formalism; neutral surface/adsorbate model.	Surface DFT calculations combined with solution-combustion experiments	Si–OH adsorption was calculated to be favorable, with an adsorption energy of approximately 1.53 eV, and was related to crystal growth and oxygen-reduction behavior. The model describes surface incorporation rather than equilibrium bulk silicate substitution.	Solution-combustion synthesis, XRD/Rietveld refinement, SEM, and FTIR.	[37]
Oxygen species	Oxygen inserted into thermally generated hydroxyl-channel vacancies; 1 × 1 × 4 vacancy-rich models	Pre-existing OH vacancy and hydroxyl channel	Filling of OH vacancies under non-equilibrium plasma conditions	Neutral vacancy-containing supercell/slab models; no explicit charged-defect correction reported.	Periodic DFT and NEB migration calculations; high-performance plasma experiments	Calculated oxygen/hydroxyl migration barriers were approximately 1 eV. Plasma-generated fields and energetic species were proposed to enable oxygen penetration, although the plasma itself was not explicitly simulated.	XPS, Raman, EIS, NMR, and CO_2_ conversion experiments; plasma treatment increased conductivity and catalytic product yield.	[38]

Abbreviations: DFT, density functional theory; DOS, density of states; EPR, electron paramagnetic resonance; GGA, generalized gradient approximation; HAp, hydroxyapatite; MD, molecular dynamics; NEB, nudged elastic band; PAW, projector augmented wave; PBE, Perdew–Burke–Ernzerhof functional; PW91, Perdew–Wang 1991 functional; XRD, X-ray diffraction.

**Table 3:** Representative computational studies of co-doped hydroxyapatite.

Dopant Pair	Model/Composition	Preferred Site(s)	Relative Arrangement	Charge Compensation	Defect Charge Treatment	Method	Interaction/Binding Energy	Type of Co-Doping Effect	Main Conclusion	Experimental Validation	Ref.
Mn^2+^/NO_3_^−^	88-atom monoclinic HAp; Mn substitution combined with B-type nitrate and a Ca vacancy	Isolated Mn slightly preferred Ca(II); nitrate changed the local preference toward nearby Ca(I)	Most stable Mn at the nearest Ca(I), approximately 0.34 nm from nitrate N	Ca vacancy compensating NO_3_^−^ for PO_4_^3−^ substitution	Neutral compensated defect complex; nitrate substitution compensated by a Ca vacancy.	Quantum ESPRESSO; PBE+U with (U = 3) eV; selected 176-atom checks	Approximately −0.30 eV for nearest Ca(I); −0.15 eV for nearby Ca(II); interaction weakened with distance	Energetic association and site-preference reversal	Nitrate altered the preferred Mn position. Stabilization was attributed mainly to compensation of local lattice distortions rather than direct magnetic interaction.	Pulsed EPR relaxation supported non-random spatial association, although the exact pair distance was obtained from DFT rather than direct EPR distance measurements.	[39]
Mg^2+^/CO_3_^2−^	88-atom monoclinic HAp; A- and B-type carbonate with Mg at Ca(I) or Ca(II)	Mg at Ca(II) for A-type carbonate	Mg adjacent to carbonate in the OH channel; Mg–O approximately 1.98 Å	A-type: intrinsically charge balanced in the selected model; B-type models also included Na^+^	Charge-neutral coupled models; B-type carbonate models additionally included Na^+^ compensation.	Quantum ESPRESSO; PW91; ultrasoft pseudopotentials	−0.308 eV for Mg+ A-type CO_3_^2−^; approximately −0.037 to −0.076 eV for B-type arrangements	Direct cation–anion complex and selective defect stabilization	Mg selectively stabilized A-type carbonate through direct coordination; Mg at remote Ca(I) provided essentially no energetic gain.	Consistent with earlier FTIR observations showing an increased relative population of A-type carbonate in Mg-containing HAp.	[40]
Sr^2+^/F^−^	18 unit-cell models with 10–100 mol.% Sr and 50–100% replacement of OH sites by F	Sr site depended on concentration; F occupied OH sites	Selected F substitutions introduced into the lowest-energy Sr-HAp configurations	Not required for either isovalent substitution	Neutral isovalent substitutions; no charged-defect correction required.	CASTEP; GGA-PBE; full cell relaxation	Not explicitly calculated as a Sr–F pair-binding energy	Global structural stabilization and functional complementarity	F made Sr-containing models more stable and reduced Sr-induced lattice expansion, but a discrete Sr–F complex was not demonstrated.	XRD/Rietveld and biological assays; F contributed antibacterial activity, while Sr promoted osteogenic differentiation.	[41]
Mg^2+^/Zn^2+^	Unit-cell models with four Mg/Zn site combinations; experimental 5Mg5Zn and 10Mg10Zn compositions	Isolated Mg: Ca(I); isolated Zn: Ca(II); lowest tested binary configuration placed both at Ca(I)	Both dopants at Ca(I) in the lowest calculated binary model	Not required; both are divalent	Neutral isovalent co-substitution; no charged-defect correction required.	VASP; spin-polarized GGA-PBE DFT	Not reported; all calculated formation energies were positive	Configurational redistribution and functional complementarity	Independently determined single-dopant preferences were not additive in the binary system. At high experimental loading, HAp transformed to a whitlockite-like phase.	XRD, TEM, compositional analysis, MC3T3 assays, and antibacterial testing; moderate co-doping combined osteogenic and antibacterial effects.	[42]
Sr^2+^/Zn^2+^	Unit-cell models containing Sr and Zn at Ca(I) and/or Ca(II); porous scaffold experiments	Both dopants favored Ca(II) among the tested configurations	Lowest calculated model contained Sr and Zn at Ca(II) sites	Not required; both are divalent	Neutral isovalent co-substitution; no charged-defect correction required.	CASTEP-based periodic DFT and formation-energy comparisons	Not explicitly reported; spread between tested codoped configurations approximately 0.257 eV	Structural compensation and functional complementarity	Sr-induced expansion and Zn-induced contraction partly cancelled in the average lattice parameters. Positive formation energies indicated endothermic substitution.	XRD revealed secondary Zn-containing calcium-phosphate phases; Sr supported osteogenesis and Zn antibacterial activity.	[44]
Ce^3+^/Sm^3+^	Constant 0.4 at.% Ce with 0–1.8 at.% Sm in assumed periodic compositions	Not systematically determined	Predefined periodic dopant arrangements	Not explicitly modelled despite trivalent-for-divalent substitution	Charge compensation not explicitly modelled; no charged-defect correction reported.	Periodic DFT band-structure and DOS calculations	Not calculated	Assumed-composition electronic tuning	Calculated band gaps decreased from 4.6078 to 4.0477 eV with increasing Sm, but no defect association, site landscape, or explicit charge-compensation mechanism was evaluated.	XRD, SEM, thermal analysis, and compositional measurements; actual incorporation of both dopants was lower than nominal.	[45]
Pr^3+^/Ce^3+^	Constant 0.35 at.% Ce with 0.35–1.40 at.% Pr; assumed periodic compositions	Not systematically determined	Predefined Pr/Ce arrangements	Not explicitly modelled	Charge compensation not explicitly modelled; no charged-defect correction reported.	Quantum ESPRESSO-based structural and electronic calculations	Not calculated	Assumed-composition electronic and optical tuning	The reported band gap decreased from 4.5156 to 4.3097 eV. The calculation characterized the selected compositions but did not establish mutual affinity or a charge-neutral defect complex.	XRD and FTIR showed HAp as the major phase, but β-TCP increased from approximately 3.4 to 5.9% with Pr addition.	[47]
La^3+^/Mg^2+^	Fixed Mg content with increasing La concentration in modelled and synthesized HAp	Not systematically determined	Predefined La/Mg distributions	Compensation for La^3+^ → Ca^2+^ not explicitly resolved	La^3+^ compensation not explicitly resolved; no charged-defect correction reported.	CASTEP-based band-structure and DOS calculations	Not calculated	Functional co-doping without demonstrated energetic synergy	The calculated gap decreased moderately from approximately 4.58 to 4.44 eV, but the models did not establish preferred dopant positions or a coupled compensation mechanism.	XRD, EDX, thermal, magnetic, and cell-viability measurements; biological and structural trends were composition dependent.	[51]

Abbreviations: DFT, density functional theory; DOS, density of states; EPR, electron paramagnetic resonance; GGA, generalized gradient approximation; HAp, hydroxyapatite; MD, molecular dynamics; NEB, nudged elastic band; PAW, projector augmented wave; PBE, Perdew–Burke–Ernzerhof functional; PW91, Perdew–Wang 1991 functional; XRD, X-ray diffraction.

## Data Availability

The original contributions presented in this study are included in the article. Further inquiries can be directed to the corresponding author.
